# Global, regional, and national burden of ischemic heart disease attributed to non-optimal temperature, 1990–2021: an age-period-cohort analysis of the global burden of disease study

**DOI:** 10.3389/fcvm.2025.1559432

**Published:** 2025-11-14

**Authors:** Xiaoqing Xia, Deji Suona, Jing Yu, Hong Zhi, Lina Wang

**Affiliations:** 1 Key Laboratory of Environmental Medicine Engineering, Ministry of Education, Department of Epidemiology & Biostatistics, School of Public Health, Southeast University, Nanjing, China; 2 Department of Cardiology, ZhongDa Hospital, Southeast University, Nanjing, China

**Keywords:** burden of disease, IHD, non-optimal temperature, age-period-cohort model, mortality

## Abstract

**Background:**

In recent years, non-optimal temperature has significantly impacted global health including ischemic heart disease (IHD).

**Methods:**

Data regarding the burden of IHD caused by non-optimal temperature were sourced from the Global Burden of Disease Study 2021. Temporal trends of the age-standardized mortality rate (ASMR) and the age-standardized disability-adjusted life years rate (ASDR) were estimated by annual percentage change (EAPC) from 1990 to 2021. Age-period-cohort modeling was employed to investigate IHD-related mortality.

**Results:**

The number of IHD deaths and DALYs resulting from non-optimal temperature experienced a rise of 71.6% and 60.6%, respectively. And it showed regional imbalances: in the region with low-middle socio-demographic index (SDI), it was increased [EAPC for ASMR: 0.39% (95% CI: 0.2%, 0.58%), EAPC for ASDR: 0.33% (95% CI: 0.15%, 0.52%)], while decreased in the high SDI region, [EAPC for ASMR: −3.44% (95%CI: −3.58%, −3.3%), EAPC for ASDR: −3.18% (95%CI: −3.32%, −3.03%)]. APC modeling showed that the global risk of death from IHD caused by non-optimal temperature was increased with age and was generally higher in males than in females. The period effect revealed a higher mortality risk in low-middle and low SDI regions, whereas the birth cohort effect indicated a lower mortality risk in high-middle and high SDI regions.

**Conclusions:**

The IHD burden caused by non-optimal temperature significantly varied according to the genders, SDI regions, and countries from 1990 to 2021. It is crucial to implement effective strategies to mitigate the impact of non-optimal temperature on IHD, particularly among men, the elderly, and the lower SDI regions.

## 1 Introduction

Ischemic heart disease (IHD), chiefly resulting from coronary artery disease and myocardial infarction, is marked by high mortality and disability rates, rendering it the cardiovascular condition with the most substantial disease burden (1). According to the latest Global Burden of Disease (GBD) report, it was estimated that around 182 million disability-adjusted life years (DALYs) due to IHD were experienced worldwide in that year (2), and it will cause 10.8 million deaths in 2040 and will continue to represent a substantial hazard to global cardiovascular health, leading to considerable medical burden and economic pressure (3). Therefore, it is of critical importance to implement effective strategies for the control of global non-communicable diseases, with particular attention to IHD (4).

In recent years, extreme heat waves and cold waves have become more frequent around the world, which directly endanger the health of individuals (5). A study covering 27 countries on five continents revealed that heat and cold caused 2.2 and 9.1 excess fatalities per 1,000 cardiovascular deaths, respectively (6). The adverse consequences of extreme temperature on agricultural workers in nations with a medium and low human development index can lead to a significant reduction in working hours, which in turn can have a damaging impact on the economic stability of vulnerable groups (5). Epidemiological evidence indicates that the relationship between temperature and mortality is inverted J-shaped, particularly concerning cardiovascular diseases (CVD), with IHD exhibiting the highest attributable mortality fraction (7). According to previous research, the influence of extreme cold on IHD mortality tends to persist over a longer period, whereas the impact of extreme heat is typically more acute and transient (6, 8). Notwithstanding the acknowledgment of the substantial burden imposed by IHD and the escalating global exposure to extreme climates, there is presently no comprehensive study quantifying the IHD burden caused by non-optimal temperature from a global perspective.

Based on the GBD 2021 database, this study analyzed data from 204 countries and regions to thoroughly evaluate the influence of non-optimal temperature exposure on the burden of IHD. Utilizing the Estimated Annual Percentage Change (EAPC) and the Age-Period-Cohort (APC) model enables us to discern temporal trends and decompose mortality risk data across three dimensions: age, period, and cohort within various SDI regions. The study aims to provide reliable information to help governments formulate scientific public health interventions and policies to reduce the harm to cardiovascular health caused by frequent extreme temperature around the world.

## 2 Materials and methods

### 2.1 Data sources

The GBD 2021 study employs the latest epidemiological data and standardized methodologies from 1990 to 2021 to comprehensively evaluate health losses associated with 371 diseases, injuries, and 88 risk factors among diverse age and sex groups in 204 countries and regions (2). The number of deaths, age-standardized mortality rates (ASMR), DALYs, and age-standardized DALY rates (ASDR) attributed to non-optimal temperature related to IHD between 1990 and 2021, categorized by gender, age, region, and country, were derived from the GBD 2021 (https://vizhub.healthdata.org/gbd-results/), and further categorized into five quintiles of Socio-Demographic Index (SDI). The criteria of SDI assessment included average educational attainment, fertility rates, and a composite evaluation of economic development across different nations. Each cause of death was mapped to the GBD cause of death categories by the ICD-10 coding system. The ICD-10 codes for IHD-related causes of death include I20-I21.6, I21.9-I25.9, and Z82.4-Z82.49.

The non-optimal temperature dataset was derived from the ERA5 reanalysis, a comprehensive meteorological dataset produced by the European Centre for Medium-Range Weather Forecasts (ECMWF), offering a highly detailed global atmospheric record with excellent spatial and temporal resolution (9). Exposure to non-optimal temperature is defined as encountering ambient temperature, either above or below the threshold linked to the lowest mortality risk, within the same day (10). The theoretical minimum risk exposure level (TMREL) for temperature refers to the daily temperature at which the mortality rate is lowest for all cause-specific diseases in a given location and year. It accounts for the varying exposure-response relationships across different annual temperature zones, as well as changes in disease composition over time and space (11). Within the Comparative Risk Assessment (CRA) framework, the GBD study estimated the population attributable fraction (PAF) for non-optimal temperature using the TMREL (12). To quantify the burden of IHD attributed to non-optimal temperature, the GBD study multiplied the total IHD burden by the corresponding PAFs across GBD regions, years, age groups, and sexes.

### 2.2 Statistical analysis

This study aimed to estimate the IHD burden resulting from non-optimal temperature between 1990 and 2021, using deaths, DALYs, ASMR, ASDR, and percentage changes in age-standardized rates (ASRs). EAPC, a widely used and concise metric for assessing trends in ASRs over a given period, was employed to quantify trends within the specified time interval (13). The general regression model and the EAPC calculation are presented in Equations 1, 2, respectively. The EAPC is calculated by fitting the natural logarithm of the rate to a regression model with time as the variable, generating a straight line from the log-transformed observations, with the value derived from the slope (14).

y=α+βx+ε (1)

EAPC=100×(exp(β)−1) (2)

The APC model was utilized to assess the separate impact of age, period, and birth cohort on IHD mortality associated with non-optimal temperature. The age effect reveals biological and sociological changes that occur with aging. The period effect reflects temporal events and changes impacting mortality from IHD due to non-optimal temperature. The cohort effect highlights generational variations in mortality, shaped by differing levels of exposure to risk factors. Drawing on data from GBD 2021, we obtain global and region-specific IHD mortality estimates across various SDI classifications, along with the corresponding demographic data, as the APC model's inputs. Subsequently, the derived data were tabulated, comprising the following: (a) 16 age groups, ranging from 20 to 24 to 95+ years, with consecutive age intervals of 5 years; (b) six consecutive 5-year calendar periods from 1992 to 1996 to 2017–2021; (c) twenty-one consecutive 5-year birth cohorts, from 1895 to 1899 to 1995–1999. The central birth cohort (1945–1949) and the central calendar period (2002–2006) were used as reference points to determine the period rate ratios (RRs). This study utilized the web-based APC analysis tool developed by the National Cancer Institute of the United States. (http://analysistools.nci.nih.gov/apc/) (15). The APC model generates outputs including the net drift, which reflects the overall annual percentage change while accounting for variations across periods and cohorts. For various age groups and birth cohorts, local drift logarithmically represents the yearly percentage change. The influence of age on the increasing trend of IHD is illustrated by the longitudinal age curve, which reflects the age-specific rate of the control cohort adjusted for period bias. The period RR denotes the risk relative to the reference period after adjusting for age and non-linear cohort effects. Similarly, the cohort RR indicates the relative risk of a birth cohort in comparison to the reference birth cohort, after adjusting for age and non-linear period effects. The statistical analysis and visualization presented in this article were conducted using R software (version 4.4.1). A *p*-value of less than 0.05 (two-sided) was regarded as statistically significant.

## 3 Results

### 3.1 Trends in the global IHD burden caused by non-optimal temperature

**Figure 1:**
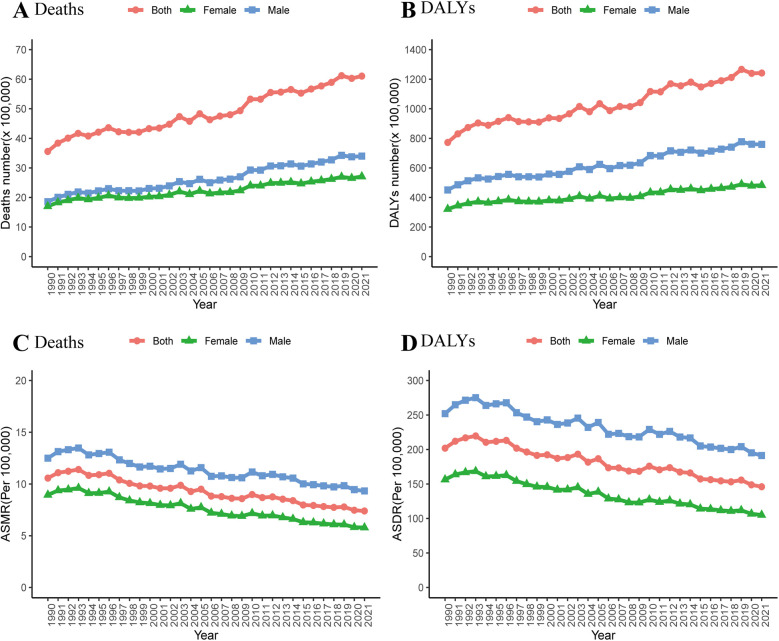
Changes in the deaths (**A,C**) and DALYs (**B,D**) attributable to non-optimal temperature globally and in different genders from 1990 to 2021.

Globally, deaths and DALYs from IHD attributable to non-optimal temperature have risen significantly over the past 32 years (Figure 1). Specifically, the number of IHD deaths due to non-optimal temperature rose from 355,690 (95% UI: 291,440–465,060) in 1990 to 610,520 (95% UI: 459,420–862,750) in 2021. Additionally, DALYs increased from 7,720,580 (95% UI: 6,227,790–10,156,650) in 1990 to 12,418,520 (95% UI: 9,158,610–17,640,350) in 2021 (Table 1). However, from 1990 to 2021, the ASMR and ASDR declined by 30.1% and 27.7%, respectively. Similarly, the absolute burden of IHD attributable to low temperature increased, whereas the ASMR and ASDR declined (Supplementary Figure S1).

**Table 1:** IHD burden and mortality attributable to non-optimal temperature in 1990 and 2021 and the temporal trends from 1990 to 2021.

Characteristics	1990				2021				EAPC (1990–2021)	
	Deaths	ASMR	DALYs	ASDR	Deaths	ASMR	DALYs	ASDR	ASMR	ASDR
	NO. × 10^3^ (95%UI)	NO. × 10^−5^ (95%UI)	NO. × 10^3^ (95%UI)	NO. × 10^−5^ (95%UI)	NO. × 10^3^ (95%UI)	NO. × 10^−5^ (95%UI)	NO. × 10^3^ (95%UI)	NO. × 10^−5^ (95%UI)	No. (95% Cl)	No. (95% Cl)
Global	355.69 (291.44∼465.06)	10.57 (8.63∼13.74)	7,720.58 (6,227.79∼10,156.65)	201.94 (164.76∼265.82)	610.52 (459.42∼862.75)	7.39 (5.57∼10.44)	12,418.52 (9,158.61∼17,640.35)	146.03 (107.55∼207.43)	−1.34% (−1.44∼−1.24)	−1.24% (−1.35∼−1.13)
Sex										
Male	185.68 (150.37∼244.73)	12.50 (10.22∼16.41)	4,508.11 (3,596.47∼5,973.28)	251.98 (202.89∼332.76)	339.8 (255.3∼473.53)	9.33 (7.05∼12.98)	7,589.53 (5,526.06∼10,668.88)	191.30 (140.47∼268.49)	−1.08% (−1.19∼−0.97)	−1.06% (−1.16∼−0.95)
Female	170.01 (137.75∼220.71)	8.95 (7.26∼11.57)	3,212.47 (2,588.8∼4,240.02)	156.44 (126.48∼206.42)	270.73 (203.91∼380.65)	5.79 (4.35∼8.14)	4,828.98 (3,550.23∼6,840.55)	105.05 (76.94∼149)	−1.63% (−1.73∼−1.53)	−1.5% (−1.61∼−1.40)
SDI										
High SDI	118.03 (100.41∼154.88)	10.75 (9.12∼14.12)	2,151.26 (1,862.97∼2,817.80)	196.60 (170.05∼257.76)	99.83 (79.89∼136.2)	4.25 (3.43∼5.82)	1,683.16 (1,372.57∼2,319.19)	83.20 (66.68∼116.00)	−3.44% (−3.58∼−3.30)	−3.18% (−3.32∼−3.03)
High-middle SDI	111.04 (96.12∼141.14)	13.43 (11.58∼17.12)	2,286.65 (1,977.35∼2,908.87)	244.78 (210.67∼311.09)	174.25 (140.2∼241.84)	9.07 (7.30∼12.61)	3,059.32 (2,474.47∼4,230.96)	158.14 (127.62∼218.78)	−1.70% (−1.93∼−1.47)	−1.93% (−2.21∼−1.66)
Middle SDI	65.83 (48.1∼88.91)	8.06 (5.83∼10.80)	1,641.97 (1,189.92∼2,220.21)	161.04 (117.42∼217.83)	184.32 (137.99∼251.71)	7.85 (5.93∼10.74)	3,827.65 (2,845.28∼5,241.03)	147.98 (110.75∼202.53)	0% (−0.13∼0.14)	−0.25% (−0.37∼−0.14)
Low-middle SDI	47.96 (28.87∼68.69)	9.00 (5.48∼12.90)	1,295.94 (778.24∼1,863.20)	202.03 (121.50∼289.48)	124.31 (74.28∼186.37)	9.53 (5.74∼14.23)	3,141.20 (1,853.5∼4,707.17)	211.78 (125.6∼317.34)	0.39% (0.20∼0.58)	0.33% (0.15∼0.52)
Low SDI	12.35 (7.84∼17.92)	6.35 (4.02∼9.13)	335.10 (211.95∼484.42)	142.43 (90.24∼206.68)	27.32 (17.63∼38.68)	6.41 (4.17∼9.00)	698.30 (451.66∼995.45)	133.91 (86.51∼190.05)	0.25% (−0.04∼0.54)	−0.11% (−0.35∼0.13)
GBD region										
Central Asia	10.96 (8.9∼15.24)	26.65 (21.58∼37.22)	231.00 (190.16∼318.57)	510.32 (420.81∼706.16)	15.56 (12.32∼22.31)	23.49 (18.71∼33.58)	323.99 (253.39∼467.55)	427.50 (336.5∼614.86)	−0.97% (−1.25∼−0.68)	−1.25% (−1.58∼−0.91)
Central Europe	23.61 (20.25∼30.37)	17.67 (15.14∼22.78)	473.47 (406.02∼606.13)	330.87 (283.81∼423.71)	23.44 (19.37∼32.75)	9.90 (8.19∼13.84)	379.07 (315.3∼529.42)	169.64 (141.02∼236.74)	−2.76% (−3.00∼−2.52)	−3.09% (−3.34∼−2.85)
Eastern Europe	49.83 (44.66∼62.63)	20.45 (18.15∼25.74)	987.81 (898.19∼1,241.35)	374.77 (338.40∼471.43)	61.74 (51.24∼84.01)	17.28 (14.33∼23.52)	1,102.11 (913.70∼1,495.09)	316.14 (262.11∼429.18)	−1.41% (−1.90∼−0.92)	−1.53% (−2.09∼−0.97)
Australasia	2.94 (2.61∼3.51)	12.96 (11.41∼15.47)	54.19 (48.78∼64.13)	233.33 (209.32∼276.36)	1.98 (1.64∼2.36)	3.22 (2.71∼3.82)	29.30 (25.33∼34.55)	53.37 (46.49∼62.93)	−4.90% (−5.06∼−4.74)	−5.15% (−5.33∼−4.97)
High-income Asia Pacific	9.44 (7.97∼11.73)	5.40 (4.49∼6.71)	171.88 (147.39∼216.29)	90.52 (77.28∼113.56)	11.64 (8.90∼14.87)	1.93 (1.56∼2.44)	167.20 (136.32∼210.61)	35.18 (29.40∼43.97)	−3.22% (−3.42∼−3.01)	−3.00% (−3.16∼−2.84)
High-income North America	47.97 (38.94∼66.47)	13.22 (10.75∼18.32)	860.71 (721.7∼1,185.27)	246.24 (207.19∼338.88)	38.89 (30.30∼54.87)	5.52 (4.35∼7.79)	670.10 (543.14∼937.92)	104.15 (84.69∼145.12)	−3.29% (−3.48∼−3.10)	−3.21% (−3.40∼−3.03)
Western Europe	57.54 (49.45∼71.64)	9.70 (8.32∼12.08)	1,011.63 (886.92∼1,255.78)	175.96 (154.35∼218.31)	36.46 (29.05∼45.62)	3.17 (2.58∼3.94)	526.33 (438.53∼650.89)	53.60 (45.86∼65.69)	−4.17% (−4.36∼−3.98)	−4.38% (−4.56∼−4.19)
Andean Latin America	0.85 (0.68∼0.97)	4.62 (3.70∼5.28)	19.01 (15.17∼21.92)	90.55 (72.23∼103.93)	1.53 (1.21∼1.91)	2.70 (2.14∼3.37)	30.39 (23.78∼37.8)	51.26 (40.25∼63.8)	−2.17% (−2.60∼−1.74)	−2.28% (−2.71∼−1.84)
Caribbean	0.51 (0.32∼0.68)	2.16 (1.42∼2.87)	10.28 (6.03∼14.03)	40.48 (24.33∼55.02)	0.68 (0.53∼0.87)	1.24 (0.96∼1.59)	13.44 (10.29∼17.29)	24.91 (19.07∼32.06)	−2.28% (−2.85∼−1.70)	−2.01% (−2.60∼−1.43)
Central Latin America	3.83 (3.25∼4.62)	5.41 (4.59∼6.54)	84.33 (71.12∼102.02)	102.63 (86.87∼124.09)	11.19 (9.61∼13.14)	4.70 (4.03∼5.52)	221.14 (191.64∼260.09)	88.95 (76.95∼104.67)	−0.61% (−0.95∼−0.26)	−0.68% (−1.03∼−0.32)
Southern Latin America	5.07 (4.6∼5.89)	12.06 (10.86∼14.03)	100.90 (92.65∼117.58)	225.13 (206.28∼262.36)	3.57 (3.15∼4.00)	3.95 (3.50∼4.44)	65.89 (59.48∼73.34)	75.78 (68.34∼84.27)	−3.22% (−3.44∼−3.01)	−3.21% (−3.41∼−3.01)
Tropical Latin America	4.11 (3.07∼5.25)	5.16 (3.84∼6.58)	100.7 (75.28∼129.12)	108.99 (81.21∼139.42)	5.75 (4.36∼7.37)	2.29 (1.73∼2.93)	129.83 (100.01∼166.31)	50.21 (38.56∼64.33)	−3.08% (−3.41∼−2.76)	−3.05% (−3.38∼−2.71)
North Africa and Middle East	35.32 (24.16∼50.52)	25.26 (17.37∼35.9)	887.80 (607.19∼1,284.41)	521.60 (356.9∼748.41)	73.54 (49.33∼111.85)	19.25 (13.23∼28.87)	1,728.40 (1,137.64∼2,670.27)	380.10 (253.27∼583.53)	−1.10% (−1.23∼−0.97)	−1.27% (−1.39∼−1.15)
Southeast Asia	3.88 (1.71∼5.81)	1.77 (0.78∼2.69)	101.18 (42.81∼151.75)	38.24 (16.67∼57.54)	11.71 (8.33∼15.93)	2.10 (1.48∼2.89)	272.73 (193.85∼367.42)	42.32 (30.13∼57.53)	0.29% (−0.14∼0.73)	0.06% (−0.39∼0.50)
South Asia	49.03 (26.23∼71.94)	9.56 (5.08∼14.12)	1,388.95 (749.47∼2,021.09)	223.47 (119.74∼327.07)	144.16 (78.93∼211.4)	10.70 (5.88∼15.64)	3,689.07 (2,004.72∼5,441.98)	241.79 (131.82∼355.78)	0.66% (0.39∼0.94)	0.49% (0.24∼0.75)
East Asia	45.22 (33.73∼61.02)	7.45 (5.56∼10.11)	1,096.10 (816.62∼1,474.58)	137.25 (102.61∼185.30)	156.81 (117.23∼218.38)	8.52 (6.38∼11.91)	2,778.55 (2,060.4∼3,880.65)	139.41 (103.29∼194.75)	0.87% (0.52∼1.23)	0.41% (0.11∼0.71)
Oceania	0.12 (0.09∼0.16)	4.52 (3.46∼5.98)	3.63 (2.67∼4.89)	109.62 (81.87∼145.93)	0.27 (0.20∼0.36)	4.00 (3.02∼5.25)	8.02 (5.89∼10.67)	94.80 (70.58∼125.77)	−0.45% (−0.75∼−0.14)	−0.50% (−0.80∼−0.20)
Central Sub-Saharan Africa	0.35 (−0.18∼0.6)	2.04 (−1.01∼3.42)	9.05 (−4.75∼15.77)	41.9 (−21.29∼71.76)	0.72 (0.37∼1.06)	1.74 (0.89∼2.55)	18.50 (9.37∼27.5)	34.88 (17.82∼51.02)	−1.39% (−1.90∼−0.87)	−1.46% (−1.98∼−0.95)
Eastern Sub-Saharan Africa	1.96 (1.48∼2.43)	3.05 (2.35∼3.85)	52.97 (39.56∼66.21)	67.48 (51.27∼84.14)	3.89 (3.05∼4.96)	2.79 (2.16∼3.55)	100.83 (78.08∼129.15)	57.37 (44.88∼73.34)	−0.76% (−0.98∼−0.55)	−1.04% (−1.25∼−0.82)
Southern Sub-Saharan Africa	1.27 (1.03∼1.51)	5.39 (4.33∼6.48)	31.33 (25.9∼37.23)	112.3 (92.44∼133.69)	2.64 (2.29∼3.01)	5.56 (4.8∼6.33)	61.72 (53.84∼71.00)	109.15 (95.22∼124.96)	−0.16% (−0.61∼0.30)	−0.33% (−0.8∼0.15)
Western Sub-Saharan Africa	1.90 (0.05∼3.63)	2.62 (0.10∼4.95)	43.64 (−0.61∼85.48)	51.63 (0.43∼99.62)	4.38 (1.78∼7.24)	2.83 (1.17∼4.69)	101.91 (40.02∼169.62)	54.01 (21.81∼89.37)	0.22% (−0.12∼0.56)	0.08% (−0.26∼0.43)

No., number; DALYs, disability-adjusted life-years; ASMR, age-standardized mortality rate; ASDR, age-standardized DALY rate; UI, uncertainty interval; EAPC, estimated annual percentage change; CI, confidential interval; SDI, sociodemographic index.

Between 1990 and 2021, the global IHD burden caused by non-optimal temperature was markedly higher in men compared to women. In 2021, the number of deaths among men reached 339,800 (95% UI: 255,300–473,530), exceeding that of women, which stood at 270,730 (95% UI: 203,910–380,650) (Table 1). Similarly, DALYs among men reached 7.59 million, which was 1.57 times higher than those in women (4.83 million). Over the past three decades, both the ASMR and ASDR of IHD attributable to non-optimal temperature have consistently been higher in males than in females (Figure 1).

**Figure 2:**
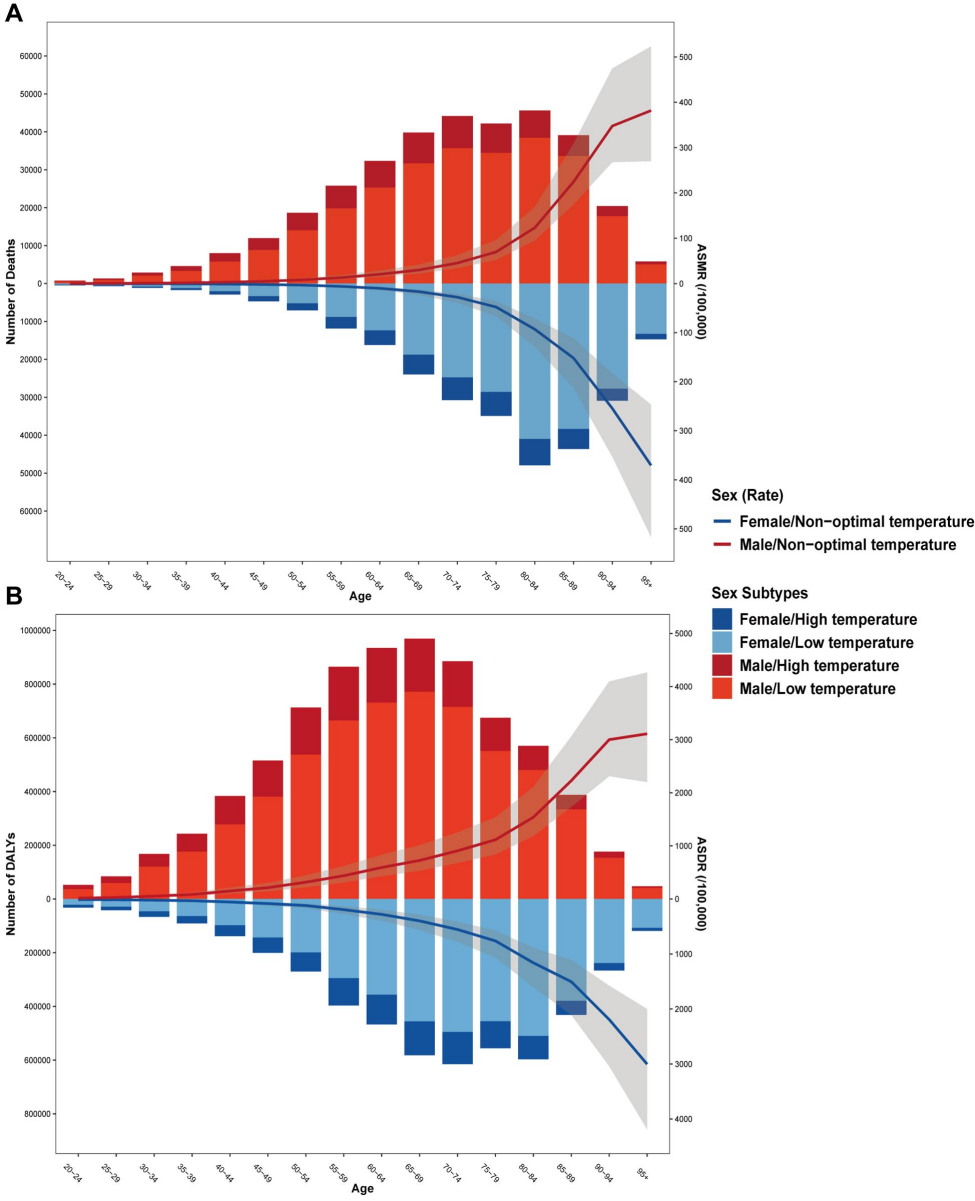
The age distribution of IHD deaths **(A)** and DALYs **(B)** attributable to non-optimal temperature. The bar was the number of IHD deaths and DALYs attributable to non-optimal temperature. The line represents the age-specific mortality rate and DALY rate attributable to non-optimal temperature.

As illustrated in Figure 2, the number of deaths and DALYs attributed to non-optimal temperature exhibited an initial increase followed by a decline with advancing age for both genders. Both males and females demonstrated a pronounced growth in ASMR and ASDR after the age of 70, with low temperature emerging as the dominant factor driving this rise. Most fatalities occurred among individuals aged 55–89, with the 80–84 age group experiencing the highest death toll (Figure 2A). Additionally, the 65–69 age group accounted for the largest number of DALYs, with the majority of DALYs concentrated in the 50–84 age range (Figure 2B).

### 3.2 The IHD burden resulting from non-optimal temperature in various regions and countries

**Figure 3:**
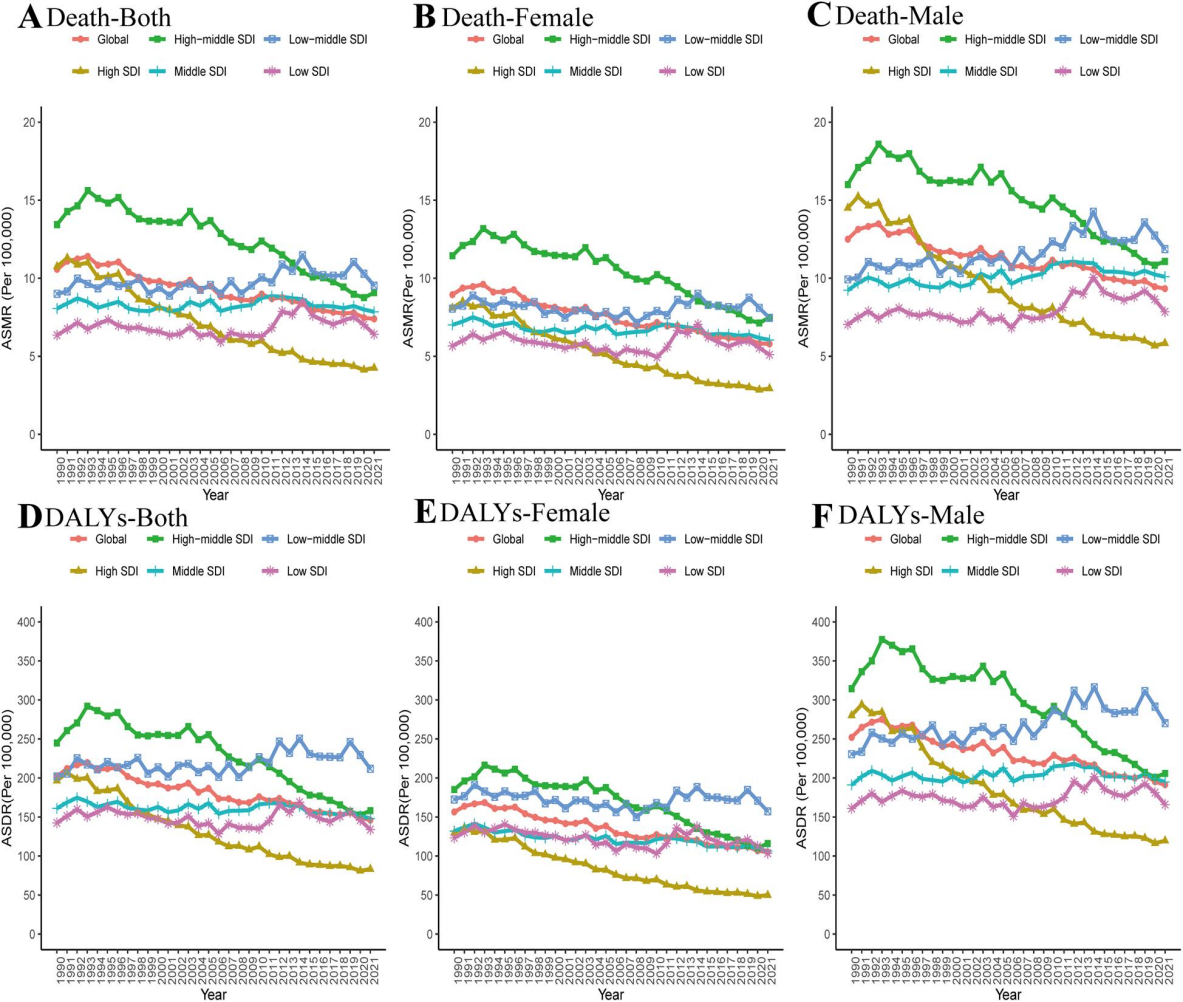
Changes in the ASMR (**A–C**) and ASDR (**D–F**) of IHD attributable to non-optimal temperature globally and in different SDI regions from 1990 to 2021.

In 2021, the low-middle SDI region presented the highest ASMR and ASDR, while the high SDI region recorded the lowest ASMR and ASDR (Table 1). From 1990 to 2021, across all SDI regions, only non-optimal temperature in the high and high-middle SDI regions demonstrated decreasing trends in both ASMR and ASDR for IHD (Figures 3A,D). Similar patterns were observed in ASMR and ASDR when gender stratification was compared across all SDI regions. Additionally, males exhibited higher ASMR and ASDR than females (Figures 3B,C,E,F and Table 1). Similarly, in all SDI regions, males bore a higher burden of IHD attributable to low temperature than females (Supplementary Figure S2).

**Figure 4:**
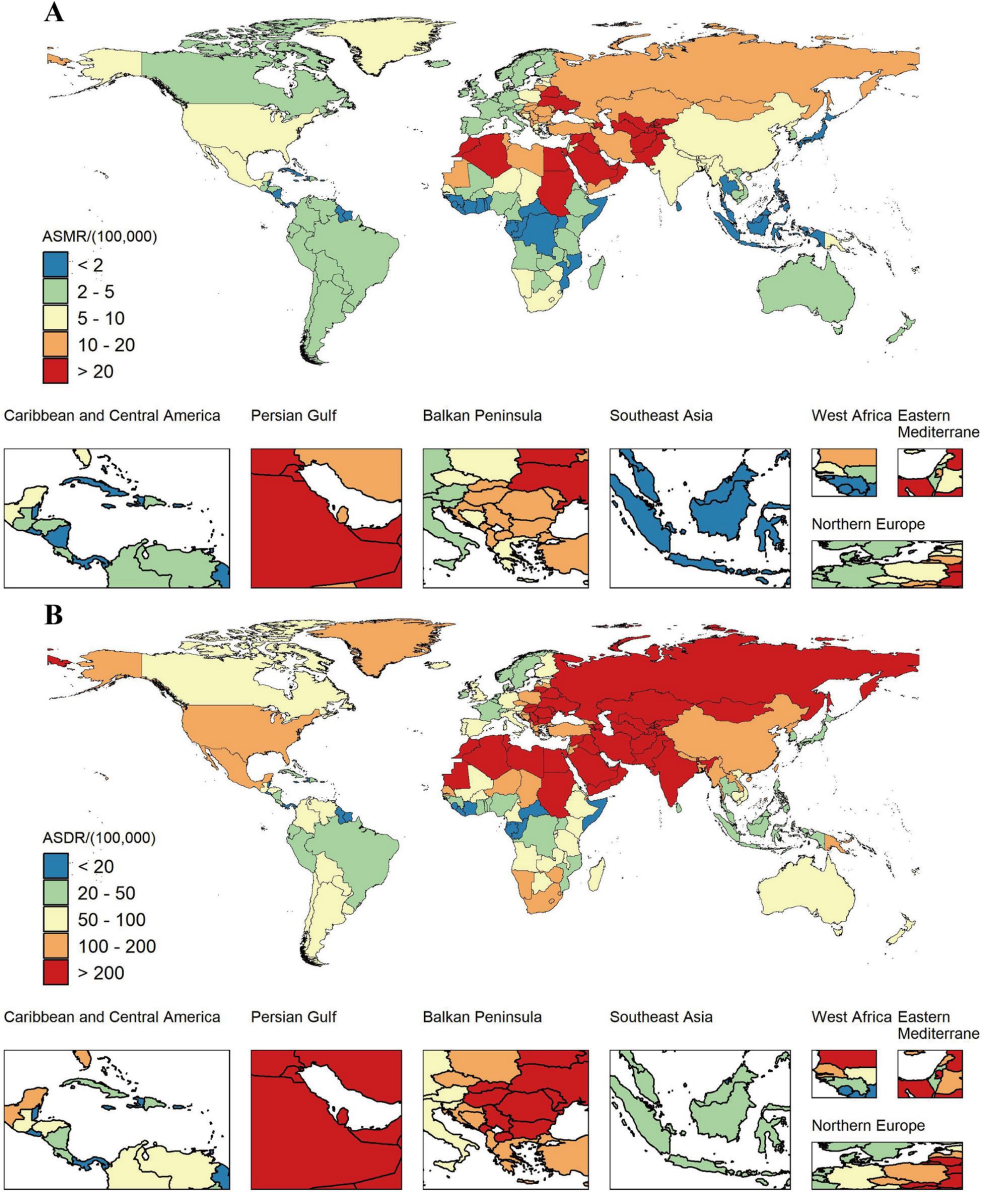
The spatial distribution of IHD ASMR **(A)** and ASDR **(B)** attributable to non-optimal temperature for both genders in 2021. Maps from: “Global national administrative boundary data”, Resource and Environmental Science Data Platform (https://www.resdc.cn/).

In terms of the 21 GBD regions, the IHD burden caused by non-optimal temperature in 2021 was highest in Central Asia (ASMR: 23.49; ASDR: 427.5), followed by North Africa and the Middle East (ASMR: 19.25; ASDR: 380.1), with Eastern Europe ranking third (ASMR: 17.28; ASDR: 316.14) (Table 1). However, in 2021, the Caribbean region exhibited the lowest ASMR and ASDR at 1.24 and 24.91, respectively, followed by Sub-Saharan Central Africa (ASMR: 1.74; ASDR: 34.88) and the high-income Asia-Pacific (ASMR: 1.93; ASDR: 35.18). China and India recorded the highest number of IHD deaths and DALYs linked to non-optimal temperature in 2021, with estimates reaching 0.15 million (95% UI: 0.11, 0.21) and 2.75 million (95% UI: 1.54, 3.95), respectively (Supplementary Figure S3 and Supplementary Table S1), a situation largely contributed to by their large populations. The three countries with the highest IHD ASMR attributable to non-optimal temperature were Iraq, Turkmenistan, and Uzbekistan, with corresponding estimates of 46.26 (95% UI: 19.42, 76.04), 39.47 (95% UI: 27.50, 59.90), and 34.17 (95% UI: 25.84, 50.85), respectively. Distributed evenly across 204 countries, the top three non-optimal temperature-related ASDR were Iraq, Turkmenistan, and Uzbekistan, similar to ASMR (Figure 4 and Supplementary Table S1).

**Figure 5:**
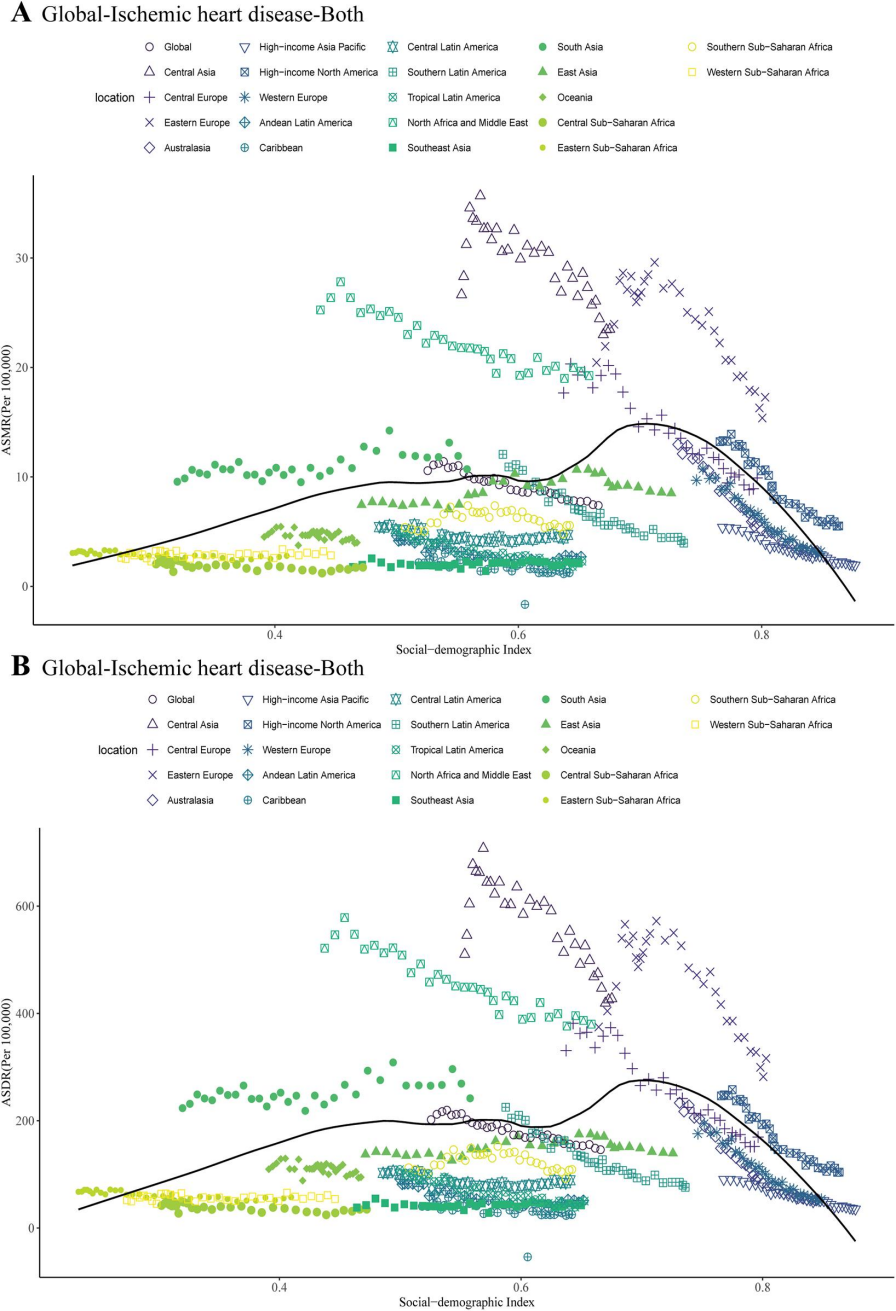
ASMR **(A)** and ASDR **(B)** attributable to non-optimal temperature across 21 geographical GBD regions by the SDI for both sexes combined from 1990 to 2021.

### 3.3 Longitudinal trends in global non-optimal temperature IHD burden

Between 1990 and 2021, the global absolute number of deaths and DALYs attributable to IHD linked to non-optimal temperature has risen by 71.6% and 60.9%, respectively. However, the global ASMR decreased by 30.1%, with an EAPC of −1.34% (95% CI: −1.44%, −1.24%), while the ASDR declined by 27.7%, with an EAPC of −1.24% (95% CI: −1.35%, −1.13%) (Table 1). Both sexes exhibited declining ASMR and ASDR from IHD attributable to non-optimal temperature. However, the decline was more substantial among females, with an EAPC of −1.63% (95% CI: −1.73%, −1.53%) for ASMR and −1.50% (95% CI: −1.61%, −1.40%) for ASDR (Table 1).

The ASMR and ASDR of IHD due to non-optimal temperature exhibited varied patterns across different SDI areas between 1990 and 2021. The region with low-middle SDI demonstrated the most pronounced increase, with EAPCs of 0.39% (95% CI: 0.20%, 0.58%) for ASMR and 0.33% (95% CI: 0.15, 0.52) for ASDR. Nevertheless, the sharpest decline occurred in high SDI region, with EAPCs reaching −3.44% (95% CI: −3.58%, −3.3%) for ASMR and −3.18% (95% CI: −3.32%, −3.03%) for ASDR (Table 1).

Between 1990 and 2021, the IHD burden resulting from non-optimal temperature exhibited a declining tendency in the majority (17 out of 21) of geographic super-regions, with the 95% CI of the EAPC falling below 0. Australasia experienced the most notable decline, with EAPCs of −4.90% (95% CI: −5.06%, −4.74%) and −5.15% (95% CI: −5.33%, −4.97%) for ASMR and ASDR, respectively. This was followed by Western Europe, with an EAPC of −4.17% (95% CI: −4.36%, −3.98%) for ASMR and −4.38% (95% CI: −4.56%, −4.19%) for ASDR. The ASMR and ASDR across four regions exhibited an increase (The 95% CIs for EAPCs exceeded 0), with the largest increases in East Asia [EAPC: 0.87% (95% CI: 0.52%, 1.23%) and 0.41% (95% CI: 0.11%, 0.71%), respectively], followed by South Asia [EAPC: 0.66% (95% CI: 0.39%, 0.94%) and 0.49% (95% CI: 0.24%, 0.75%), respectively] (Table 1).

### 3.4 Association between SDI and IHD burden caused by non-optimal temperature

We investigated the association between ASMR and SDI of IHD caused by non-optimal temperature in 21 GBD regions between 1990 and 2021. The results revealed an N-shaped association between regional SDI and the corresponding ASMR throughout the overall period. When the SDI was below 0.71, the ASMR increased gradually with the rising SDI levels, then declined significantly. From 1990 to 2021, the ASMR of IHD due to non-optimal temperature in five GBD regions, including Central Asia and Eastern Europe, exceeded expectations. In the majority of other regions, where the ASMR of IHD resulting from non-optimal temperature fell below the projections based on the SDI (Figure 5A). A similar pattern was observed for ASDR associated with IHD resulting from non-optimal temperature across diverse SDI levels (Figure 5B).

In exploring the association between the burden of IHD attributable to low temperature and SDI, both ASMR and ASDR in the 21 GBD regions exhibited a gradual increase with rising SDI levels, followed by a marked decline (Supplementary Figure S4).

### 3.5 Effects of local drift, age, period, and cohort on the burden of IHD due to non-optimal temperature

**Figure 6:**
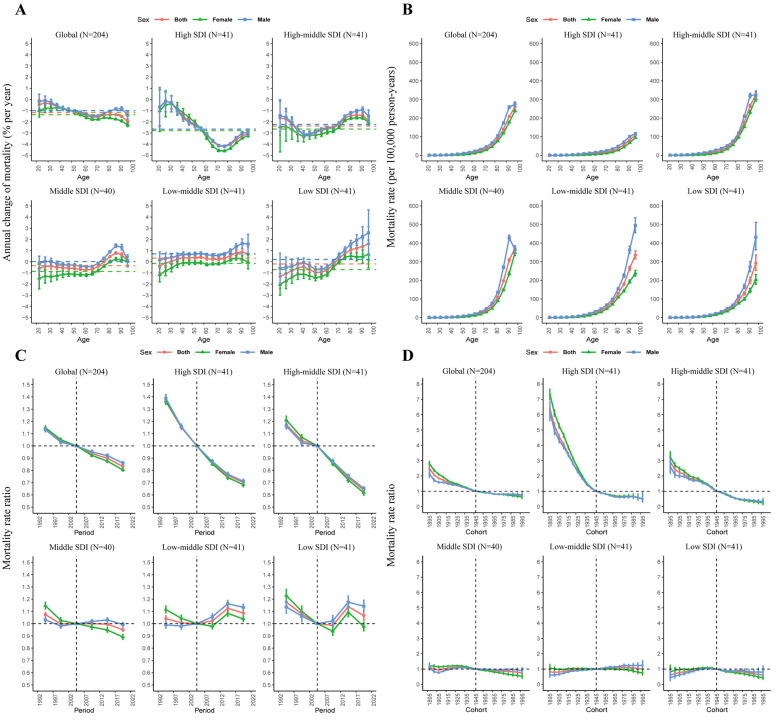
The local drifts **(A)**, age effects **(B)**, period effects **(C)**, and cohort effects **(D)** of IHD-related mortality attributable to non-optimal temperature in the global and different SDI regions from 1990 to 2021.

A net drift in IHD mortality of −1.17% per year (95% CI: −1.21, −1.13) was estimated globally using the APC model, with −2.38% (95% CI: −2.49, −2.26) in high-middle SDI region and −2.66% (95% CI: −2.77, −2.55) in high SDI region. In contrast, in low-middle SDI region, there was an upward trend in the net drift of IHD mortality (0.33%, 95% CI: 0.25, 0.41). Local drift curves for various age groups in high and high-middle SDI areas showed decreasing mortality rates across all age groups. In comparison to females, males showed smaller declines or greater increases in mortality rates across different age groups in all SDI regions (Figure 6A).

The age effect elucidated variations in risk across different age cohorts, with a consistent pattern observed globally and across all SDI regions, where risk escalated with the age increasing (Figure 6B). The period effect revealed a declining trend in mortality risk across all regions except for low and low-middle SDI areas (Figure 6C). Birth cohort effect indicated that the risk of mortality from IHD caused by non-optimal temperature declined globally. Notably, high SDI and high-middle SDI regions exhibit a downward trend, especially high SDI region, while the birth cohort effect in other regions has remained largely unaltered (Figure 6D).

Applying the APC model to the burden of IHD attributable to low temperature, we found that net drift declined across all regions. In the period effect, the risk remained stable in low and low-middle SDI regions, distinct from the patterns observed in the analysis of non-optimal temperature. The other effects were generally consistent (Supplementary Figure S5).

## 4 Discussion

### 4.1 Main findings

The findings of this study indicate that, from 1990 to 2021, the global deaths and DALYs from IHD attributable to non-optimal temperature increased substantially, whereas the corresponding ASRs showed a declining trend. In 2021, low temperature was the predominant contributor to the IHD burden attributable to non-optimal temperature, accounting for 82.8% of deaths and 80.2% of DALYs. Additionally, disparities in deaths and DALYs were evident among various age groups, predominantly affecting the middle-aged and elderly populations. Male mortality exceeded that of females, with an increase in mortality observed as age progresses. The impact of IHD resulting from non-optimal temperature was minimal in Australasia, a region characterized by a high SDI, and most pronounced in Central Asia, an area with a middle SDI, in 2021. Finally, age and period effects revealed that the risk of IHD mortality associated with non-optimal temperature was consistently heightened in low and low-middle SDI areas.

It is well-established that non-optimal temperature constitutes a risk factor for numerous diseases and poses a serious threat to human health (10, 16, 17). A study conducted in the US demonstrated that abnormal environmental temperature may exert a direct influence on the incidence of CVD and an indirect influence on CVD mortality through physical activity (18). A study involving 27 countries and 567 cities revealed that non-optimal temperature contributed to a 1% increase in mortality from IHD, with a projected increase in risk attributed to climate change (6). Moreover, a three-stage modeling study demonstrated that cold temperatures accounted for the majority of excess deaths, with mortality attributable to cold being approximately ten times higher than that associated with heat (19). Several physiological mechanisms have been put forth to explain the association between non-optimal temperature and the occurrence of IHD. These mechanisms range from cold-induced arterial vasoconstriction, which triggers increased cardiac output and fibrinogen levels, to diminished intravascular volume and dehydration during extreme heat, all contributing to a heightened risk of IHD events (20–22). Evidence has indicated that the lagged impact of low temperature on disease burden is more pronounced, and low temperature can exacerbate the adverse cardiovascular effects of nitrogen oxides (10, 23). Therefore, it is imperative to adapt global public health strategies to mitigate the adverse cardiovascular effects associated with non-optimal temperatures. Accelerating the transition to renewable energy, enhancing urban green infrastructure, and establishing early warning systems for extreme temperature events are critical measures to regulate global temperature changes and protect cardiovascular health (6, 24).

The impact of non-optimal temperature on IHD burden is significantly greater in males compared to females, with a 1.5-fold higher incidence rate of IHD in males (25). Research carried out in China revealed that, after controlling for long-term trends in seasonality, relative humidity, and other confounding factors, males exhibited a higher daily YLL due to cardiovascular diseases compared to females (26). Risk behaviors, including smoking and alcohol consumption, are more prevalent in males and may interact with non-optimal temperature exposure to elevate the risk of cardiovascular events (27). Due to occupational demands, Men are more likely than women to engage in manual outdoor labor such as construction and farming, which increases their exposure to non-optimal temperature risk factors (28). Biomarkers regulating vascular inflammation and adipokines are upregulated to a greater extent in women than in men. Furthermore, endogenous estrogen levels can diminish the risk of CVD in premenopausal women (29). Globally, the IHD burden caused by non-optimal temperature is greater among older populations compared to younger ones. Prior research has shown that older individuals are more sensitive to temperature fluctuations (16, 17). A report from Zhejiang Province, China, indicated that individuals aged 65 and older are more sensitive to both low and high temperature than those aged 0–64 (30). Consistent with this study, the IHD burden resulting from non-optimal temperature peaks in the age group of 65 and older.

Globally, the period and cohort RRs of IHD burden due to non-optimal temperature are on a downward trend. This may be attributed to recent advancements in IHD treatment techniques, such as the rapid development of thrombolysis and interventional therapies, along with growing public awareness of cardiovascular health (31–33). Furthermore, advancements in pharmaceutical technologies, coupled with global economic growth, have synergistically driven enhanced accessibility to vital myocardial infarction treatments in underdeveloped regions (34). The corresponding burden has steadily decreased in high and high-middle SDI regions in recent years, with the rate of decline having become progressively more pronounced as SDI levels rise. This may be attributed to increased access to work in climate-controlled environments and improved healthcare resources (35). Furthermore, high SDI region are more likely to have Sufficient healthcare resources and comprehensive healthcare policy frameworks. For instance, they may have effective management strategies for hypertension and promote the use of Statin medications and low-dose aspirin for prevention, thereby decreasing the IHD burden (24, 36). However, adverse period and cohort effects were persistent in areas with low-middle SDI. Surgical supply chains in healthcare facilities in low- and middle-income countries (LMICs) are inefficient and often costly, with limited access to IHD care (37). Moreover, most LMICs are situated in tropical and subtropical areas, where the occurrence of extremes is more prevalent, and heat waves may become more frequent and persist for extended durations (38). To summarize, differences in living conditions and the provision of public health care related to IHD significantly influence the variations in disease burden among nations and regions. For that reason, it is imperative to develop and implement regionally tailored global public health interventions. To evaluate the risk of IHD caused by non-optimal temperature, particularly in LMICs, it is imperative to conduct further prospective studies.

Several limitations must be noted. First, the IHD burden indicator resulting from non-optimal temperature was derived from estimates. The temperature effects were described as transient, occurring on the day of exposure, without considering lagged or cumulative impacts. The IHD burden attributed to non-optimal temperature may be underestimated due to this method. Furthermore, the ecological design of the GBD study precluded the inclusion of genetic data in our analysis. Currently, population-based epidemiological studies directly assessing the interplay between genetic factors and non-optimal temperature exposure remain limited. Future research that integrates genomic and environmental data is essential to unravel these complex relationships. Lastly, the reliance on global biodiversity data introduces variability in terms of data availability and quality across different locations. This unavoidable limitation has been acknowledged and reported in other studies, highlighting the challenges associated with data availability in this field of research (39–41).

## 5 Conclusion

Globally, the IHD burden caused by non-optimal temperature has reduced from 1990 to 2021. However, the observed trends across countries are inconsistent, with the burden shifting from high SDI nations to middle and low SDI countries, particularly among both males and the elderly. This phenomenon is driven by several factors, including increased exposure to non-optimal temperature, disparities in healthcare resources allocated to IHD care, and the effects of population growth and aging in middle and low SDI countries. These findings necessitate immediate action to enhance IHD prevention through strengthened primary health care and environmental climate management, particularly in countries and regions bearing a high disease burden and relative economic disadvantage. The findings will inform the formulation of policies for IHD prevention and climate change response in diverse geographical regions.

## Data Availability

The data employed in this study were sourced from publicly available sources: the Institute for Health Metrics and Evaluation (IHME), accessible at https://vizhub.healthdata.org/gbd-results/.
