# Supplementary material for: Global, regional, and national burden of ischemic heart disease attributed to non-optimal temperature, 1990–2021: an age-period-cohort analysis of the global burden of disease study
Source: Front Cardiovasc Med. 2025 Nov 14;12:1559432. doi: 10.3389/fcvm.2025.1559432 (PMC12661862; doi:10.3389/fcvm.2025.1559432)
Supplement: Supplementary file 1 [file Datasheet1.pdf]

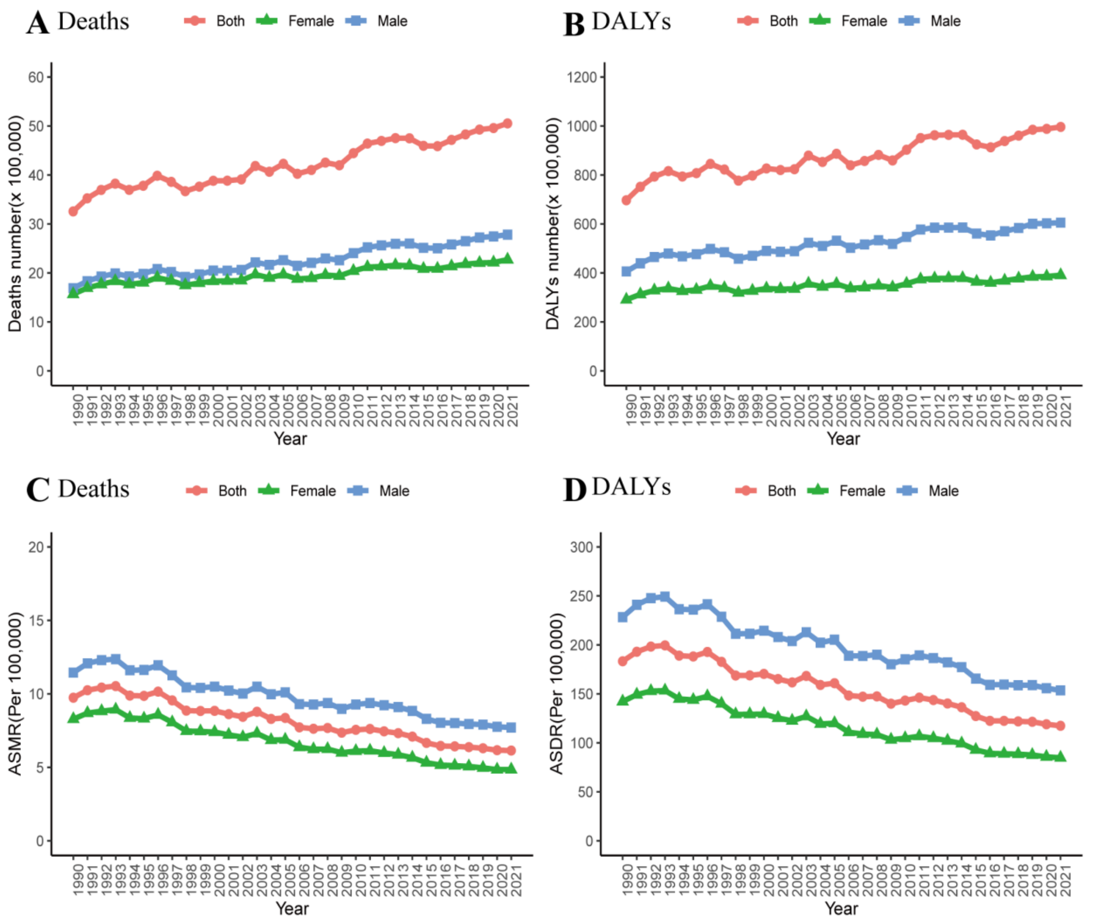

Supplementary figure 1. Changes in the deaths (**A.C**) and DALYs (**B.D**) attributable to low temperature globally and in different genders from 1990 to 2021.

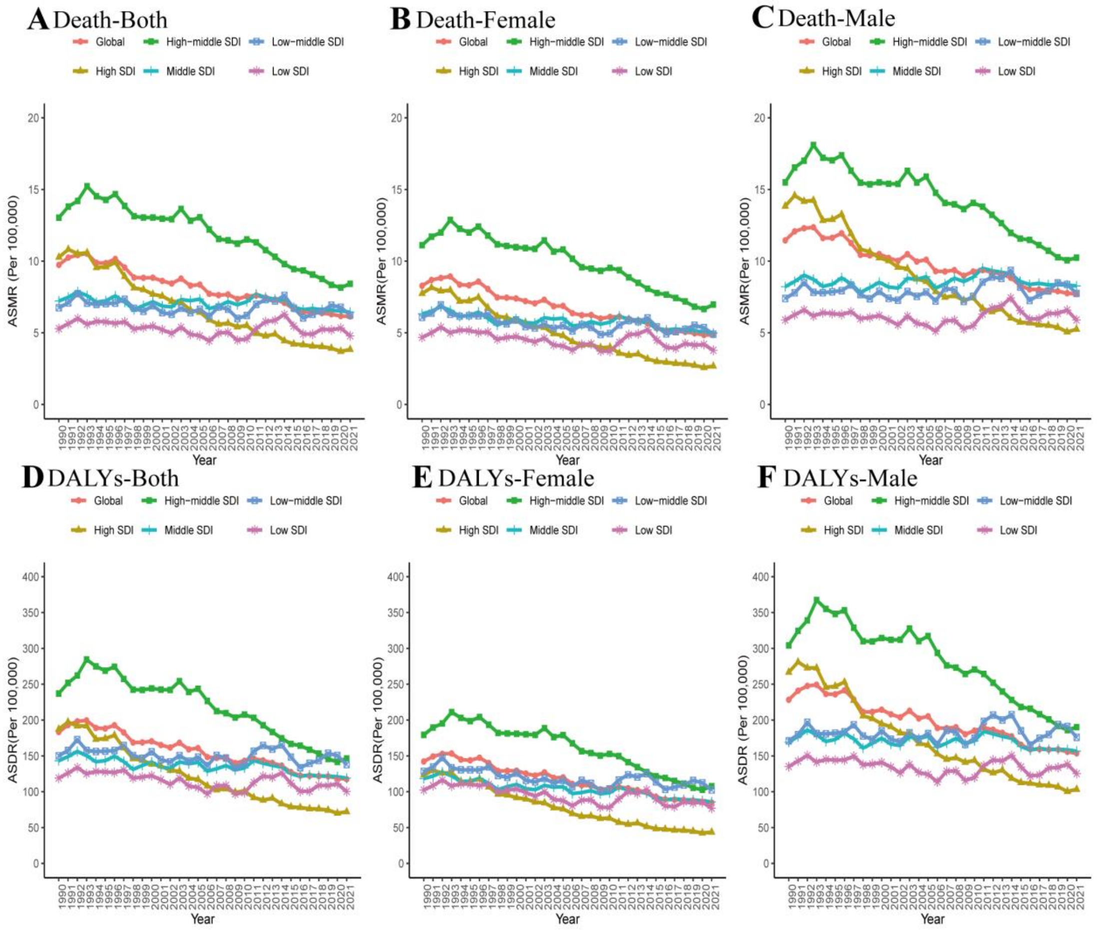

Supplementary figure 2. Changes in the ASMR (**A-C**) and ASDR (**D-F**) of IHD attributable to low temperature globally and in different SDI regions from 1990 to 2021.

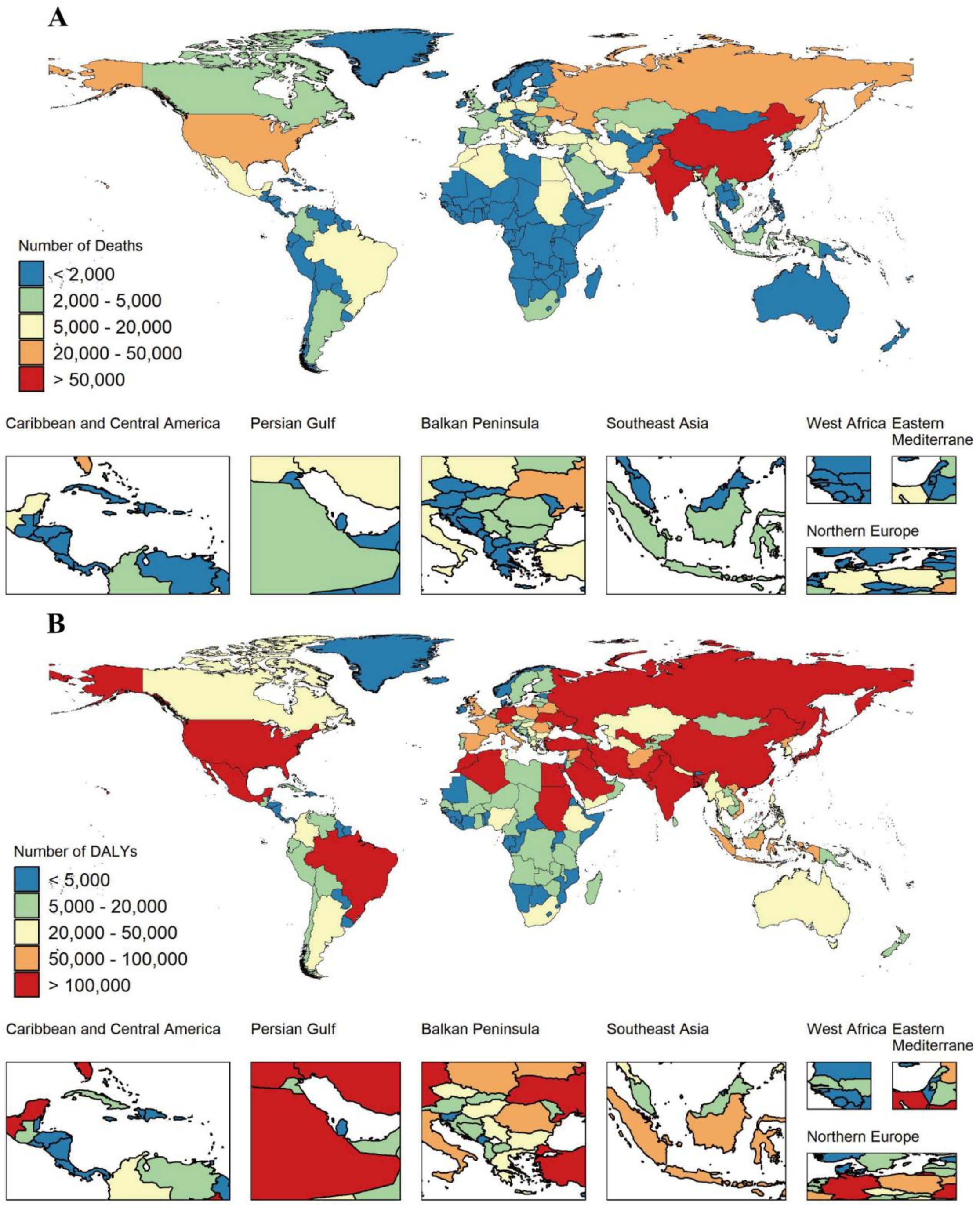

Supplementary figure 3. The spatial distribution of IHD Deaths (**A**) and DALYs (**B**) attributable to non-optimal temperature for both genders in 2021.

A Global-Ischemic heart disease-Both

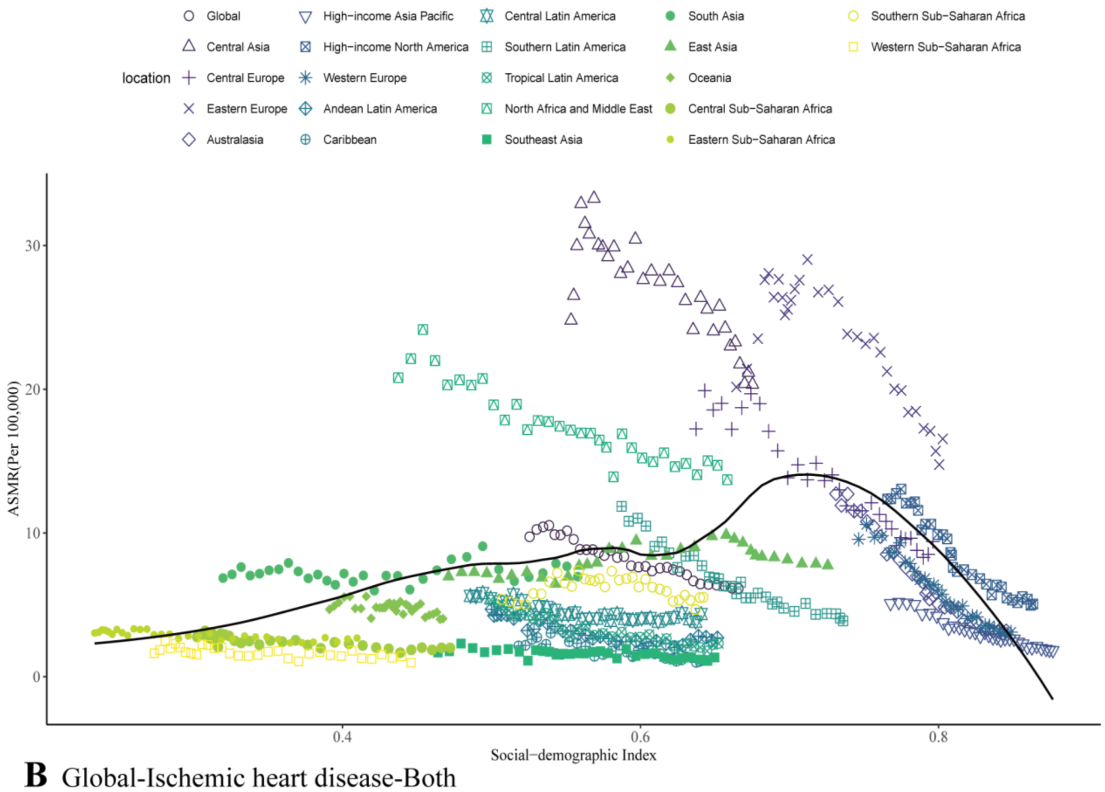

B Global-Ischemic heart disease-Both

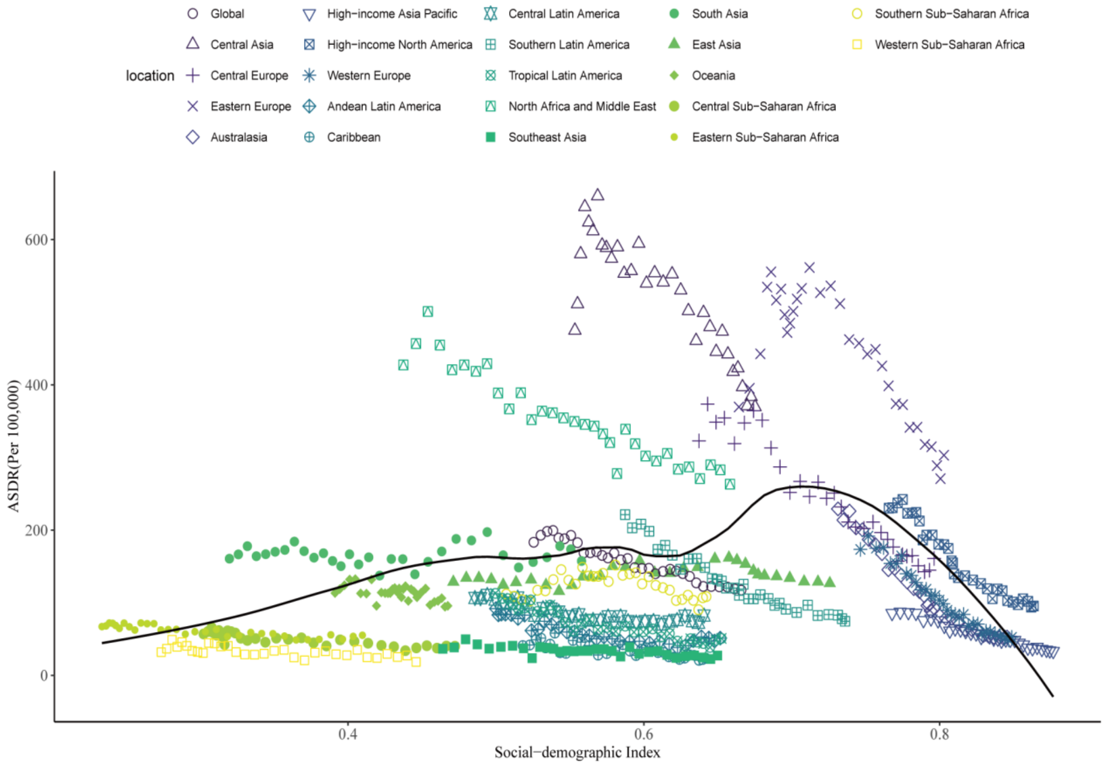

Supplementary figure 4. ASMR (**A**) and ASDR (**B**) attributable to low temperature across 21 geographical GBD regions by the SDI for both sexes combined from 1990 to 2021.

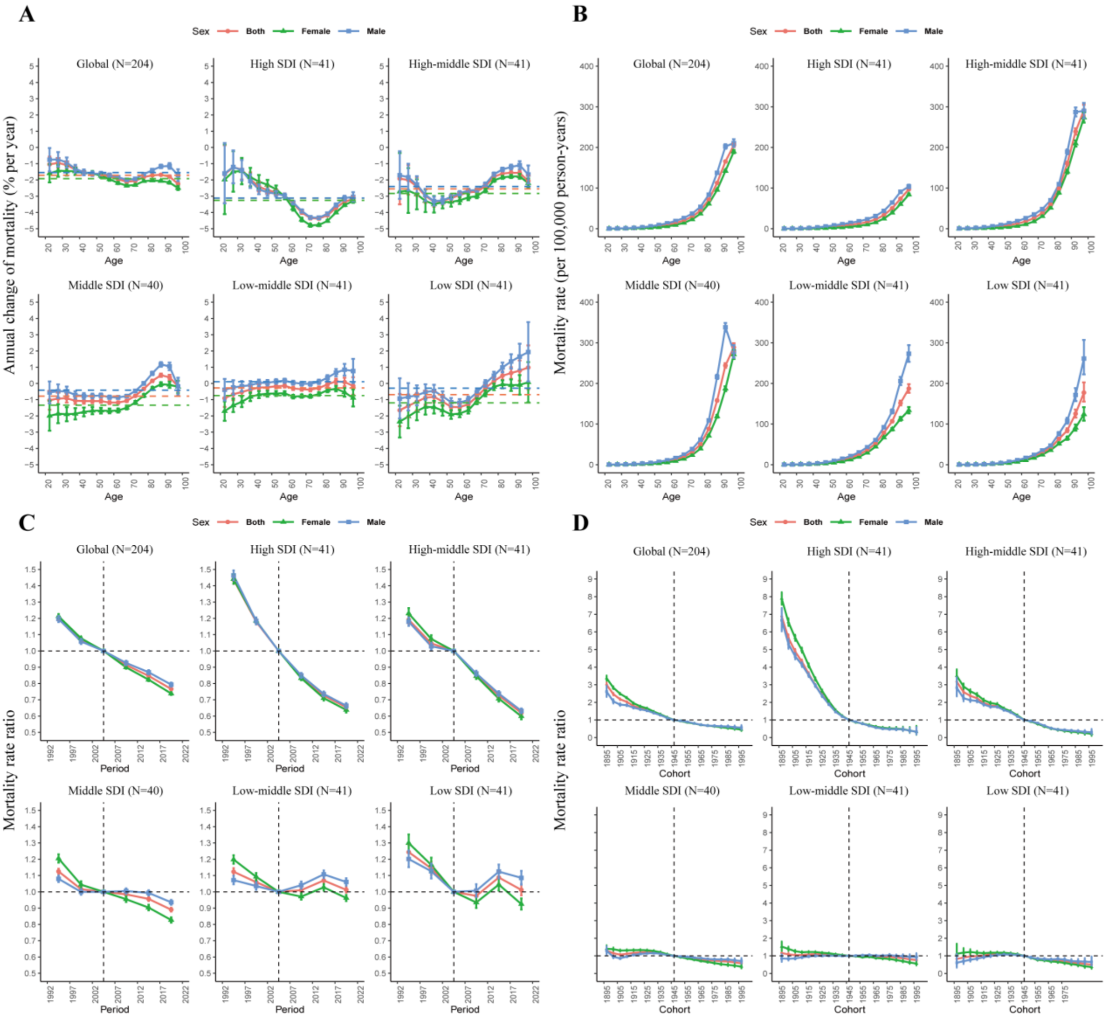

Supplementary figure 5. The local drifts (**A**), age effects (**B**), period effects (**C**), and cohort effects (**D**) of IHD-related mortality attributable to low temperature in the global and different SDI regions from 1990 to 2021.

Supplementary table 1. IHD burden and mortality attributable to non-optimal temperature in 204 countries in 2021

location	Deaths (95%UI)	DALY (95%UI)	ASMR (95%UI)	ASDR (95%UI)
Afghanistan	1855.38 (1202.21to2850.49)	53223.47 (33525.35to84523.06)	21.92 (14.53to33.17)	479.16 (309.96to736.75)
Albania	513.79 (416.86to645.08)	8323.54 (6781.43to10585.46)	12.63 (10.24to15.93)	198.39 (161.87to252.19)
Algeria	5026.03 (3718.21to7104)	100597.81 (74135.58to143876.58)	20.14 (14.99to28.26)	321.78 (237.91to456.98)
American Samoa	0.24 (0.17to0.34)	6.24 (4.3to8.74)	0.57 (0.42to0.79)	12.85 (8.96to17.93)
Andorra	4.49 (3.25to6.09)	69.08 (48.86to93.13)	2.56 (1.85to3.48)	42.69 (30.33to57.75)
Angola	248.46 (158.22to344.75)	6413.92 (4061.69to8966.34)	2.84 (1.82to3.89)	56.46 (35.87to78.24)
Antigua and Barbuda	0.22 (0.15to0.28)	4.22 (2.98to5.33)	0.24 (0.16to0.3)	4.14 (2.92to5.22)

location	Deaths (95%UI)	DALY (95%UI)	ASMR (95%UI)	ASDR (95%UI)
Argentina	2525.99 (2180.65to2915.81)	46412.75 (40929to53095.62)	4.37 (3.78to5.05)	83.05 (73.36to95.07)
Armenia	599.07 (483.32to829)	10542.04 (8402.19to14451.76)	13.93 (11.23to19.29)	246.6 (196.47to338.02)
Australia	1601.09 (1322.79to1916.15)	23522.53 (20058.5to27917.06)	3.06 (2.56to3.65)	50.7 (43.79to59.85)
Austria	1005.04 (810.37to1300.24)	13989.35 (11611.29to18015.3)	4.48 (3.67to5.77)	70.33 (59.56to90.92)
Azerbaijan	2076.97 (1673.4to2979.05)	43073.84 (34533.84to61896.06)	26.57 (21.5to37.89)	471.14 (377.8to675.98)
Bahamas	4.51 (2.78to6.51)	103.39 (63.66to149.37)	1.23 (0.76to1.77)	25.26 (15.54to36.45)
Bahrain	96.28 (47.26to149.25)	2566.24 (1251.18to3983.94)	18.74 (9.04to28.89)	315.53 (153.18to489.17)
Bangladesh	7467.31 (4086.7to11132.73)	178288.62 (96302.79to271724.01)	5.95 (3.27to8.84)	127.85 (69.27to193.01)

location	Deaths (95%UI)	DALY (95%UI)	ASMR (95%UI)	ASDR (95%UI)
Barbados	0.4 (0.25to0.55)	6.97 (4.26to9.66)	0.08 (0.05to0.11)	1.36 (0.83to1.88)
Belarus	3586.26 (2894.75to4661.43)	63297.39 (50562.97to81839.16)	21.9 (17.69to28.47)	395.62 (315.66to511.88)
Belgium	678.15 (499.29to922.94)	10130 (7721.78to13647.12)	2.39 (1.8to3.23)	41.59 (32.34to55.7)
Belize	1.38 (0.59to2.74)	30.09 (12.78to59.94)	0.52 (0.22to1.03)	10.07 (4.28to20.06)
Benin	55.65 (-88.42to118.94)	1268.93 (-2031.27to2747.56)	1.34 (-2.11to2.84)	25.43 (-40.31to54.55)
Bermuda	3.6 (2.53to5.17)	58.33 (41.29to83.62)	2.37 (1.68to3.4)	42.33 (29.88to60.57)
Bhutan	38.85 (29.47to49.57)	857.22 (625.43to1125.53)	6.9 (5.29to8.73)	139.76 (103.33to181.9)
Bolivia (Plurinational State of)	324.47 (231.82to471.74)	6872.17 (4808.21to9911.08)	4.28 (3.12to6.18)	78.77 (56.02to113.88)

location	Deaths (95%UI)	DALY (95%UI)	ASMR (95%UI)	ASDR (95%UI)
Bosnia and Herzegovina	597.33 (441.3to849.09)	10063.06 (7361.39to14258.69)	9.42 (6.93to13.42)	161.92 (117.99to229.91)
Botswana	52.13 (31.37to76.08)	1251.88 (737.11to1865.72)	4.51 (2.68to6.52)	87.94 (53to128.59)
Brazil	5518.78 (4223.03to7029.53)	124833.27 (96956.54to158132.97)	2.24 (1.72to2.86)	49.41 (38.27to62.61)
Brunei Darussalam	0.72 (0.46to0.99)	19.83 (12.46to27.3)	0.27 (0.17to0.37)	5.44 (3.48to7.43)
Bulgaria	2078.77 (1566.43to3030.36)	36660.83 (27748.35to53788.45)	14.87 (11.23to21.61)	275.45 (208.65to404.95)
Burkina Faso	334.71 (158.88to630.78)	7407.61 (3316.7to14036.77)	4.48 (2.2to8.38)	84.76 (39.61to159.84)
Burundi	126.93 (89.06to178.58)	3455.13 (2430.02to4928.79)	3.18 (2.24to4.43)	66.65 (46.64to94.06)
Cabo Verde	18.73 (13.72to25.23)	355.77 (255.89to484.5)	4.53 (3.33to6.12)	81.77 (59.14to111.34)

location	Deaths (95%UI)	DALY (95%UI)	ASMR (95%UI)	ASDR (95%UI)
Cambodia	363.29 (86.54to520.33)	8830.59 (2108.4to12762.45)	3.69 (0.88to5.34)	73.2 (17.5to104.55)
Cameroon	275.83 (164.57to465.79)	6700.65 (3919.61to11519.58)	2.82 (1.69to4.66)	54.85 (32.65to92.81)
Canada	2517.99 (2081.05to3161.26)	39075.28 (33830.7to48717.92)	3.1 (2.59to3.89)	53.51 (46.92to66.52)
Central African Republic	-0.8 (-66.81to36.14)	-22.19 (-1947.84to1058.9)	-0.05 (-3.96to2.17)	-1.05 (-84.71to46)
Chad	243.51 (69.52to512.87)	5923.9 (1701.42to12490.83)	5.23 (1.5to10.86)	104.39 (29.85to219.26)
Chile	747.04 (625.06to869.49)	14493.55 (12207.4to16761.05)	2.86 (2.4to3.33)	57 (48.02to65.9)
China	153587.19 (114657.8to213452.87)	2710087.34 (2014521.66to3774895.63)	8.72 (6.52to12.17)	141.49 (105to197.45)
Colombia	2109.62 (1716.05to2644.63)	38072.15 (30880.33to47635.39)	3.72 (3.01to4.66)	68.36 (55.52to85.38)

location	Deaths (95%UI)	DALY (95%UI)	ASMR (95%UI)	ASDR (95%UI)
Comoros	3.46 (1.63to6.16)	82.36 (38.46to144.7)	0.83 (0.4to1.48)	16.85 (7.9to29.74)
Congo	11.98 (1.12to32.77)	308.94 (29.39to854.76)	0.59 (0.06to1.56)	11.71 (1.1to31.86)
Cook Islands	-0.08 (-0.13to-0.03)	-1.61 (-2.76to-0.57)	-0.3 (-0.52to-0.11)	-6.52 (-11.23to-2.33)
Costa Rica	116.69 (95.25to153.4)	2300.68 (1911.89to3005.81)	2.07 (1.7to2.72)	41.5 (34.58to54.19)
Côte d'Ivoire	41.67 (-126.21to110.57)	1045.51 (-3168.67to2797.69)	0.49 (-1.5to1.28)	9.55 (-28.9to25.31)
Croatia	1035.68 (793.6to1501.39)	15110.33 (11576.52to21838.57)	10.55 (8.08to15.26)	163.69 (125.36to236.31)
Cuba	341.07 (251.38to443.25)	6020.45 (4422.53to7835.53)	1.63 (1.2to2.12)	30.46 (22.37to39.65)
Cyprus	121.78 (82.21to169.15)	2071.34 (1420.15to2888.93)	7.16 (4.92to9.95)	111.43 (76.35to155.83)

location	Deaths (95%UI)	DALY (95%UI)	ASMR (95%UI)	ASDR (95%UI)
Czechia	1895.9 (1592.77to2427.34)	28645.45 (24155.75to36454.38)	8.12 (6.84to10.4)	129.62 (109.48to164.99)
Democratic People's Republic of Korea	2444.85 (1620.48to3815.88)	54470.25 (36048.71to82347.57)	8.43 (5.57to13.09)	171.78 (113.27to260.99)
Democratic Republic of the Congo	453.53 (225.76to690.46)	11560.01 (5669.99to17915.22)	1.62 (0.82to2.46)	32.58 (16.22to49.58)
Denmark	293.8 (229.9to333.28)	4436.15 (3463.18to5010.06)	2.19 (1.71to2.48)	36.85 (28.9to41.63)
Djibouti	44.49 (20.8to79.62)	1221.95 (569.36to2204.71)	9.1 (4.3to15.95)	186.44 (86.8to333.01)
Dominica	0.23 (0.17to0.3)	4.46 (3.29to5.92)	0.3 (0.22to0.39)	5.45 (4.03to7.2)
Dominican Republic	222.1 (166.17to290.18)	4855.6 (3626.3to6298.55)	2.28 (1.71to2.98)	47.96 (35.84to62.27)
Ecuador	385.55 (291.11to491.06)	7448.29 (5578.05to9772.66)	2.61 (1.98to3.29)	46.74 (35.16to60.95)

location	Deaths (95%UI)	DALY (95%UI)	ASMR (95%UI)	ASDR (95%UI)
Egypt	13270.1 (6848.06to22712.47)	345094.29 (178942.05to593877.98)	28.54 (14.65to48.68)	562.78 (290.99to960.52)
El Salvador	1.46 (-26.54to42.55)	26.31 (-497.68to795.23)	0.02 (-0.39to0.63)	0.41 (-7.85to12.49)
Equatorial Guinea	3.66 (0.58to5.88)	89.76 (14.03to149.21)	0.95 (0.15to1.53)	18.2 (2.87to29.1)
Eritrea	99.24 (60.16to155.25)	2873.15 (1701.81to4572.43)	4.53 (2.8to6.95)	96.46 (58.81to150.4)
Estonia	172.51 (138.26to212.55)	2472.57 (2032.11to3039.34)	5.28 (4.31to6.5)	85.31 (70.37to104.96)
Eswatini	31.31 (21.36to46.3)	848.85 (559.25to1268.81)	6.85 (4.86to9.74)	147.43 (100.23to217.53)
Ethiopia	1105.08 (850.12to1397.32)	27258.72 (21006.31to34515.69)	2.96 (2.26to3.76)	60.02 (46.17to75.51)
Fiji	24.05 (15.74to36.25)	652.05 (422.2to991.45)	3.75 (2.46to5.59)	83.04 (54.44to125.02)

location	Deaths (95%UI)	DALY (95%UI)	ASMR (95%UI)	ASDR (95%UI)
Finland	771.82 (632.38to970.88)	10783.3 (9130.07to13559.44)	4.91 (4.09to6.18)	79.24 (68.3to99.81)
France	3817.2 (2878.32to5145.24)	52144.81 (41035.72to69615.72)	2.04 (1.58to2.72)	34.36 (27.63to45.76)
Gabon	6.51 (2.07to12.24)	152.63 (48.84to287.84)	0.8 (0.25to1.49)	15.35 (4.87to28.85)
Gambia	26.98 (11.86to40.57)	620.32 (272.65to942.44)	3.32 (1.46to4.98)	64.59 (28.44to97.12)
Georgia	529.88 (415.7to724.42)	9859.21 (7714.82to13355.41)	8.36 (6.55to11.37)	168.63 (131.85to228.32)
Germany	9294.37 (7392.21to11786.31)	133795.13 (110853.12to169810.7)	3.93 (3.19to4.96)	65.07 (55.11to82.59)
Ghana	226.61 (76.22to355.74)	5510.86 (1866.62to8747.4)	1.72 (0.58to2.7)	33.68 (11.36to52.97)
Greece	1807.7 (1494.93to2270.71)	27909.29 (23694.75to34673.54)	6.19 (5.22to7.72)	119.66 (103.77to148.03)

location	Deaths (95%UI)	DALY (95%UI)	ASMR (95%UI)	ASDR (95%UI)
Greenland	3.51 (2.18to5.04)	82.56 (50.9to118.17)	6.15 (3.83to8.76)	121.48 (75.47to174.33)
Grenada	0.12 (0.07to0.16)	2.55 (1.53to3.4)	0.12 (0.07to0.15)	2.27 (1.37to3.03)
Guam	0.38 (-0.27to1.76)	9.61 (-6.88to45.21)	0.18 (-0.13to0.83)	4.71 (-3.37to22.26)
Guatemala	327.63 (267.33to439.1)	6625.37 (5351.91to8967.62)	3.59 (2.92to4.79)	62.35 (50.74to84.47)
Guinea	66.48 (-10.67to120.59)	1518.67 (-250.54to2755.32)	1.4 (-0.22to2.53)	27.47 (-4.47to49.85)
Guinea-Bissau	5.61 (-10.88to27.69)	152.57 (-293.12to744.03)	1 (-1.99to4.83)	20.76 (-40.31to102.03)
Guyana	0.91 (-5.04to4.3)	22.04 (-122.06to104.67)	0.16 (-0.89to0.76)	3.41 (-18.83to16.07)
Haiti	52.76 (-6.66to117.37)	1343.6 (-167.15to2995.11)	0.91 (-0.12to2.02)	18.68 (-2.35to41.47)

location	Deaths (95%UI)	DALY (95%UI)	ASMR (95%UI)	ASDR (95%UI)
Honduras	218.05 (158.75to298.68)	4591.41 (3341.33to6418.68)	4.3 (3.14to5.86)	77.56 (56.59to107.19)
Hungary	2628.39 (1925.57to3929.79)	42571.82 (30925.33to64156.46)	12.28 (8.99to18.4)	214.71 (155.69to323.3)
Iceland	28.08 (22.65to34.01)	411.22 (347.78to498.72)	4.12 (3.4to4.99)	67.83 (58.33to82.74)
India	108564.47 (60895.94to156176.23)	2749414.08 (1541404.75to3948365.86)	9.97 (5.58to14.37)	224.76 (125.98to322.58)
Indonesia	2717.65 (2102.07to3649.27)	71053.87 (54635.52to95631.65)	1.41 (1.1to1.89)	29.49 (22.73to39.38)
Iran (Islamic Republic of)	9556.02 (6623.93to14732.7)	197004.25 (135226.24to300052.14)	14.17 (9.73to21.89)	260.53 (179.35to398.69)
Iraq	8574.81 (3590.11to14175.42)	199901.41 (84748.09to336933.25)	46.26 (19.42to76.04)	876.26 (368.04to1451.74)
Ireland	250.85 (191.35to322.78)	3962.83 (3155.53to5091.74)	2.97 (2.28to3.82)	49.56 (39.75to63.58)

location	Deaths (95%UI)	DALY (95%UI)	ASMR (95%UI)	ASDR (95%UI)
Israel	279.28 (134.68to454.22)	4110.91 (1972.26to6663.85)	2 (0.96to3.25)	31.99 (15.36to51.69)
Italy	6859.09 (5374.05to8699.05)	91938.93 (74660.73to116271.72)	3.57 (2.86to4.52)	57.22 (48.49to72.6)
Jamaica	10.32 (7.09to14.58)	186.55 (130.79to266.74)	0.31 (0.21to0.44)	5.87 (4.09to8.42)
Japan	9627.34 (7419.79to11816.73)	135220.66 (111166.35to162954.56)	1.98 (1.63to2.39)	37.6 (32.76to44.64)
Jordan	414.85 (272.72to596.38)	10246.49 (6623.18to14785.85)	7.18 (4.75to10.22)	136.99 (90.05to196.76)
Kazakhstan	2365.97 (1807.56to3325.86)	45442.4 (34352.43to64427.36)	17.4 (13.4to24.37)	287.43 (218.43to405.54)
Kenya	459.76 (340.5to614.86)	11457.17 (8486.34to15364.05)	2.57 (1.9to3.46)	50.35 (37.22to67.4)
Kiribati	0.02 (-0.7to0.14)	0.55 (-21.83to4.45)	0.03 (-1.1to0.22)	0.68 (-26.41to5.39)

location	Deaths (95%UI)	DALY (95%UI)	ASMR (95%UI)	ASDR (95%UI)
Kuwait	593.84 (253.32to943.33)	15867.26 (6675to25208.27)	22.46 (9.67to35.51)	454.86 (193.22to727.92)
Kyrgyzstan	742.98 (585.15to991.57)	14939.47 (11754.04to19965.81)	20.22 (15.84to27.01)	347.69 (275.24to464.23)
Lao People's Democratic Republic	245.89 (151.94to369.2)	6318.8 (3872.77to9397.78)	6.48 (4to9.52)	133.18 (82.26to199.56)
Latvia	440.34 (375.59to532.53)	6944.47 (5926.23to8420.34)	9.6 (8.17to11.59)	170.53 (145.76to206.96)
Lebanon	418.25 (329.88to528.48)	7150.01 (5713.88to9094.3)	6.37 (5.04to8.07)	114.87 (91.88to146.3)
Lesotho	49.43 (26.25to79.09)	1249.97 (639.67to2044.99)	5.57 (3.02to8.78)	117.63 (61.93to189.78)
Liberia	3.75 (-4.74to14.25)	91.92 (-119.15to355.34)	0.23 (-0.29to0.85)	4.4 (-5.57to16.78)
Libya	655.5 (356.42to1059.68)	16386.2 (8973.41to26703.57)	14.34 (7.94to23.24)	296.55 (161.17to482.39)

location	Deaths (95%UI)	DALY (95%UI)	ASMR (95%UI)	ASDR (95%UI)
Lithuania	851.35 (718.4to1061.75)	12618.81 (10773.82to15843.84)	12.53 (10.66to15.65)	207.59 (177.26to260.06)
Luxembourg	33.13 (26.65to41.2)	499.14 (411.79to619.99)	2.75 (2.24to3.43)	44.79 (37.06to55.47)
Madagascar	410.31 (287.75to559.15)	12001.38 (8219to16482.6)	4.51 (3.16to6.13)	97.49 (68.76to132.17)
Malawi	239.52 (143.83to350.58)	6529.81 (3887.96to9522.25)	3.78 (2.29to5.56)	81.55 (49.09to118.98)
Malaysia	254.29 (210.87to385.17)	5947.24 (4965.49to9023.24)	1.01 (0.82to1.53)	20.96 (17.49to31.82)
Maldives	0.69 (0.05to1.83)	15.62 (1.1to41.41)	0.24 (0.02to0.63)	4.32 (0.3to11.44)
Mali	296.84 (129.31to587.39)	7199.16 (3120.8to14065.48)	4.27 (1.87to8.24)	83.6 (36.46to165.9)
Malta	55.13 (41.42to71.64)	844.33 (643.62to1096.17)	5.01 (3.81to6.5)	86.05 (65.88to111.16)

location	Deaths (95%UI)	DALY (95%UI)	ASMR (95%UI)	ASDR (95%UI)
Marshall Islands	0.09 (-0.07to0.28)	2.91 (-2.11to8.58)	0.31 (-0.23to0.94)	7.39 (-5.41to21.96)
Mauritania	212.07 (89.45to374.59)	4502.37 (1880.62to7966.95)	11.8 (5.02to20.81)	218.74 (91.5to387.43)
Mauritius	45.07 (31.87to63.34)	970.36 (677.23to1364.93)	2.65 (1.88to3.74)	55.65 (38.98to78.28)
Mexico	7443.99 (6287.71to8992.92)	148856.9 (125581.4to182201.8)	6.52 (5.51to7.85)	120.27 (101.3to146.77)
Micronesia (Federated States of)	0.18 (-0.15to0.55)	5.14 (-4.35to16.34)	0.28 (-0.23to0.87)	6.47 (-5.46to20.49)
Monaco	4.51 (3.52to5.62)	65.9 (52.06to82.54)	3.81 (3.01to4.73)	66.44 (52.59to84.02)
Mongolia	274 (212.34to375.31)	6111.11 (4697.2to8389.32)	16.92 (13.03to23.28)	294.67 (228.84to403.4)
Montenegro	135.86 (113.22to169.04)	2375.35 (1982to2976.63)	16.06 (13.32to19.95)	260.54 (217.3to326.71)

location	Deaths (95%UI)	DALY (95%UI)	ASMR (95%UI)	ASDR (95%UI)
Morocco	6484.39 (4824.36to8311.76)	140989.68 (102884.42to180935.27)	22.01 (16.53to28.16)	423.22 (311.68to543.37)
Mozambique	132.1 (65.2to219.91)	3517.65 (1702.35to5853.78)	1.49 (0.72to2.48)	30.81 (15.14to51.12)
Myanmar	2163.37 (1312.69to3236.5)	49502.86 (30270.17to74728.02)	5.27 (3.19to7.84)	104.48 (63.84to157.39)
Namibia	56.17 (33.25to84.23)	1353.35 (793.46to2090.95)	5.07 (2.97to7.6)	100.78 (59.6to152.26)
Nauru	0.05 (0.01to0.09)	1.54 (0.39to2.88)	0.94 (0.24to1.7)	23.17 (5.89to42.4)
Nepal	1764.29 (1279.3to2462.11)	42189.71 (30257.97to59857.96)	8.72 (6.36to12.33)	181.53 (130.71to255.32)
Netherlands	867.51 (630.85to1175.42)	12636.5 (9380.78to16888.24)	2.19 (1.6to2.96)	34.79 (26.07to46.36)
New Zealand	374.71 (304.7to458.33)	5775.48 (4776.25to7002.21)	4.07 (3.34to4.97)	67.67 (56.41to81.93)

location	Deaths (95%UI)	DALY (95%UI)	ASMR (95%UI)	ASDR (95%UI)
Nicaragua	49.7 (39.41to60.57)	1020.86 (822.06to1250.54)	1.19 (0.94to1.46)	21.56 (17.36to26.33)
Niger	398.33 (179.78to696.43)	9358.41 (4160.81to16330.31)	6.41 (2.88to11.26)	121.61 (55.26to211.49)
Nigeria	1725.55 (528.43to2941.11)	40207.14 (11519.96to70795.68)	2.4 (0.75to4.04)	45.41 (13.78to77.83)
Niue	0.06 (0.04to0.07)	1.22 (0.89to1.62)	2.7 (2.02to3.57)	57.95 (42.12to77.04)
North Macedonia	332.86 (241.92to484.38)	6248.59 (4554.58to9154.95)	13.99 (10.2to19.92)	220.44 (161.11to321.43)
Northern Mariana Islands	0.48 (0.37to0.6)	12.88 (9.99to16.06)	1.12 (0.88to1.39)	24.19 (18.84to29.98)
Norway	295.35 (249.2to337.11)	4258.63 (3728.85to4814.67)	2.49 (2.13to2.83)	40.09 (35.67to45.18)
Oman	382.41 (188.6to610.56)	9764.52 (4827.79to15668.38)	26.48 (13.22to42.12)	491.14 (241.75to782.68)

location	Deaths (95%UI)	DALY (95%UI)	ASMR (95%UI)	ASDR (95%UI)
Pakistan	26321.14 (12203.86to42758.55)	718323.31 (333671.11to1177530.08)	24.87 (11.54to40.36)	543.78 (253.15to883.97)
Palau	0.04 (-0.02to0.13)	1 (-0.49to3.56)	0.2 (-0.1to0.74)	4.5 (-2.26to16.18)
Palestine	240.03 (141.46to383.66)	5505.94 (3255.71to8793.18)	12.77 (7.57to20.23)	229.65 (135.15to366.98)
Panama	16.33 (1.4to22.26)	307.07 (26.37to418.83)	0.36 (0.03to0.48)	6.86 (0.59to9.36)
Papua New Guinea	225.93 (160.19to304.05)	6799.46 (4770.71to9267.06)	5.14 (3.7to6.89)	118.4 (84.06to159.4)
Paraguay	235.96 (110.83to382.03)	4998.91 (2376.13to8094.63)	4.3 (2.02to6.97)	85.78 (40.73to139.38)
Peru	821.55 (607.04to1077.31)	16072.2 (11901.19to21105.38)	2.44 (1.81to3.19)	46.96 (34.71to61.65)
Philippines	1228.62 (971.79to1542.16)	32349.37 (25413.84to40415.28)	1.7 (1.35to2.14)	37.59 (29.69to47.11)

location	Deaths (95%UI)	DALY (95%UI)	ASMR (95%UI)	ASDR (95%UI)
Poland	5522.42 (4647.27to7008.72)	87395.85 (75273.52to110229.18)	7.09 (6to8.99)	119.17 (103.08to149.89)
Portugal	827.51 (658.38to1001.09)	12827.65 (10342.22to15386.51)	2.85 (2.28to3.42)	52.78 (43.46to63.07)
Puerto Rico	15.41 (10.78to20.06)	254.58 (176.86to334.38)	0.18 (0.13to0.24)	3.68 (2.56to4.84)
Qatar	105.48 (42.94to182.04)	3161.65 (1252.17to5491.84)	19.92 (8.18to33.57)	333.13 (138.09to573.9)
Republic of Korea	1988.01 (1304.29to3047.46)	31437.63 (21044.41to47196.89)	2.21 (1.45to3.4)	34.91 (23.4to52.34)
Republic of Moldova	1036.13 (774.75to1493.14)	19064.25 (14363.02to27465.05)	17.15 (12.82to24.71)	321.09 (241.91to462.76)
Romania	4646.74 (3577.95to6586.52)	76877.43 (59973.81to109343.55)	11.48 (8.86to16.31)	206.24 (161.44to293.97)
Russian Federation	34844.59 (29016.74to47028.37)	645353.46 (540473.59to863829.38)	14.47 (12.05to19.54)	273.7 (229.68to366.17)

location	Deaths (95%UI)	DALY (95%UI)	ASMR (95%UI)	ASDR (95%UI)
Rwanda	157.26 (108.74to232.81)	3885.77 (2658.01to5738.68)	3.21 (2.18to4.86)	62.61 (43.24to92.74)
Saint Kitts and Nevis	0.16 (0.12to0.21)	3.57 (2.45to4.72)	0.3 (0.21to0.39)	5.46 (3.81to7.11)
Saint Lucia	0.17 (-0.66to0.25)	3.15 (-12.24to4.64)	0.07 (-0.29to0.11)	1.33 (-5.17to1.96)
Saint Vincent and the Grenadines	0.15 (0.09to0.2)	2.83 (1.62to3.86)	0.12 (0.07to0.16)	2.09 (1.2to2.85)
Samoa	2.16 (1.65to2.85)	53.99 (40.53to72.23)	1.69 (1.28to2.23)	36.81 (27.72to49.04)
San Marino	1.99 (1.35to2.87)	27.38 (17.93to40.32)	1.96 (1.29to2.88)	32.91 (21.38to48.94)
Sao Tome and Principe	0.23 (0.15to0.32)	5.25 (3.46to7.42)	0.26 (0.17to0.36)	4.86 (3.23to6.74)
Saudi Arabia	4704.65 (2072.28to7738.29)	154159.78 (67287.25to254919.26)	27.07 (12.05to44.4)	603.16 (267.33to988.88)

location	Deaths (95%UI)	DALY (95%UI)	ASMR (95%UI)	ASDR (95%UI)
Senegal	360.57 (185.91to566.86)	7987.76 (4095.42to12541.96)	5.68 (2.96to8.88)	107.51 (55.28to169.6)
Serbia	2367.74 (1738.59to3456.97)	37831.84 (27825.1to54876.62)	13.77 (10.12to20.1)	227.09 (167.9to329.58)
Seychelles	0.68 (0.53to0.86)	15.45 (11.99to19.61)	0.67 (0.51to0.83)	13.44 (10.41to17.01)
Sierra Leone	24.78 (-1.53to48.63)	584.85 (-35.79to1156.63)	0.79 (-0.05to1.58)	15.62 (-0.96to30.79)
Singapore	26.53 (11.57to32.92)	524.28 (226.92to646.59)	0.32 (0.14to0.4)	6.18 (2.68to7.63)
Slovakia	1173.73 (961.19to1570.97)	19047.49 (15489.19to25604.89)	12.39 (10.14to16.54)	201.5 (163.76to270.74)
Slovenia	164.91 (129.35to228.78)	2398.48 (1914.23to3340.72)	3.09 (2.45to4.29)	50.5 (40.4to70.65)
Solomon Islands	-0.21 (-5.38to1.71)	-6.17 (-156.95to50.22)	-0.07 (-1.8to0.57)	-1.64 (-41.2to13.11)

location	Deaths (95%UI)	DALY (95%UI)	ASMR (95%UI)	ASDR (95%UI)
Somalia	-10.98 (-89.76to50.17)	-327.28 (-2777.44to1553.43)	-0.22 (-1.76to0.96)	-4.86 (-38.99to21.84)
South Africa	2129.79 (1864.94to2450.84)	48748.11 (42854.96to56365.35)	5.48 (4.77to6.26)	107.25 (94.04to123.72)
South Sudan	114.53 (52.18to203.52)	3126.23 (1379.71to5618.43)	3.57 (1.69to6.25)	76.48 (34.86to136.16)
Spain	3485.27 (2852.25to4191.6)	52384.19 (44615.81to61861.68)	2.86 (2.4to3.4)	51.89 (45.33to60.73)
Sri Lanka	322.63 (212.29to442.95)	6993.05 (4450.41to9785.3)	1.31 (0.87to1.78)	26.58 (17.14to37.06)
Sudan	5033.5 (2629.73to8536.59)	127523.23 (63984.47to217933.39)	30.03 (15.72to50.06)	618.01 (320.79to1055.42)
Suriname	0.94 (-3.96to1.83)	21.59 (-91.13to41.85)	0.15 (-0.65to0.3)	3.37 (-14.29to6.52)
Sweden	834.21 (690.34to956.47)	11267.21 (9623.08to12875.6)	3.08 (2.59to3.51)	47.31 (40.93to53.92)

location	Deaths (95%UI)	DALY (95%UI)	ASMR (95%UI)	ASDR (95%UI)
Switzerland	602.15 (470.74to738.01)	7880.79 (6429.54to9632.3)	2.53 (2.02to3.1)	38.26 (32.06to46.54)
Syrian Arab Republic	3163.98 (2340.08to4591.29)	73409.93 (53218.6to109052.82)	31.46 (23.81to45)	589.5 (435.38to855.55)
Taiwan (Province of China)	776.83 (311.36to1291.44)	13989.82 (5686.58to23327.78)	1.77 (0.71to2.93)	33.7 (13.73to56.22)
Tajikistan	885.17 (652.37to1277.35)	19498.98 (14251.87to28084.66)	21.51 (15.79to30.98)	380.95 (279.66to549.8)
Thailand	1710.55 (844.64to2658.92)	34890.4 (17232.34to54262.22)	1.59 (0.79to2.46)	33.57 (16.65to52.26)
Timor-Leste	15.63 (10.3to21.76)	365.19 (238.65to511.07)	2.18 (1.46to3.02)	43.43 (28.6to60.18)
Togo	57.93 (-21.32to109.17)	1462.35 (-534.07to2747.64)	2 (-0.75to3.7)	39.46 (-14.55to74.11)
Tokelau	0 (-0.01to0.01)	-0.01 (-0.16to0.27)	-0.04 (-0.5to0.87)	-0.87 (-10.74to18.73)

location	Deaths (95%UI)	DALY (95%UI)	ASMR (95%UI)	ASDR (95%UI)
Tonga	0.09 (0.04to0.15)	2.02 (0.98to3.51)	0.12 (0.06to0.2)	2.49 (1.21to4.31)
Trinidad and Tobago	2.42 (-15.02to4.99)	51.13 (-315.82to105.6)	0.13 (-0.8to0.27)	2.69 (-16.61to5.56)
Tunisia	1777.46 (1144.79to2834.17)	35081.61 (22327.36to55514.78)	15.1 (9.74to23.99)	274.37 (175.76to435.7)
Turkey	8797.18 (6838.25to12066.13)	167036.63 (130584.73to230278.61)	10.47 (8.16to14.46)	184.94 (144.67to254.67)
Turkmenistan	1291.19 (892.05to1977.44)	27729.76 (19048.08to42860.62)	39.47 (27.5to59.9)	739.57 (512to1138.01)
Tuvalu	0.01 (-0.03to0.05)	0.14 (-0.88to1.28)	0.06 (-0.37to0.56)	1.38 (-8.43to12.35)
Uganda	188.82 (129.1to265.9)	4878.86 (3308.32to6826.15)	1.54 (1.07to2.19)	31.52 (21.59to44.41)
Ukraine	20804.65 (15321.96to28924.65)	352359.48 (257000.6to495813.28)	26.12 (19.24to36.35)	451.42 (328.91to638.76)

location	Deaths (95%UI)	DALY (95%UI)	ASMR (95%UI)	ASDR (95%UI)
United Arab Emirates	567.81 (257.35to923.51)	17566.12 (7949.4to28737.67)	33.01 (15.22to52.75)	560.12 (257.76to891.67)
United Kingdom	4206.15 (3180.3to5117.21)	67425.37 (52255.14to80766.59)	2.87 (2.2to3.47)	52.08 (40.61to62.07)
United Republic of Tanzania	582.17 (394.37to799.88)	14670.13 (9928.03to20371.03)	2.65 (1.8to3.64)	55.12 (37.48to75.91)
United States of America	36363.82 (28125.94to51766.7)	630935.25 (506753.37to889102.19)	5.82 (4.57to8.28)	110.32 (88.55to154.7)
United States Virgin Islands	0.58 (0.4to0.78)	10.4 (7.09to14.08)	0.34 (0.23to0.46)	6.25 (4.26to8.46)
Uruguay	293.1 (256.4to330.35)	4979.31 (4456.28to5594.31)	4.67 (4.15to5.26)	90.22 (81.6to101.43)
Uzbekistan	6795.21 (5092.35to10078.08)	146794.1 (109101.37to217173.88)	34.17 (25.84to50.85)	618.97 (462.76to916.76)
Vanuatu	3.96 (2.24to5.28)	116.69 (66.03to157.03)	2.65 (1.51to3.52)	61.35 (34.66to81.87)

location	Deaths (95%UI)	DALY (95%UI)	ASMR (95%UI)	ASDR (95%UI)
Venezuela (Bolivarian Republic of)	901.73 (681.76to1167.2)	19335.31 (14455.46to25267.44)	3.17 (2.4to4.08)	64.78 (48.52to84.45)
Viet Nam	2624.05 (1195.79to4186.37)	55099.18 (25862.01to89755.11)	3.14 (1.4to5.03)	58.13 (26.9to93.74)
Yemen	1754.14 (1268.36to2394.72)	44549.14 (31727.34to61996.04)	15.13 (11.01to20.48)	308.96 (224.05to424.6)
Zambia	231.7 (131.28to356.48)	6108.25 (3333.21to9450.51)	4.16 (2.43to6.4)	85.2 (48.16to131.14)
Zimbabwe	323.09 (207.79to465.98)	8265.46 (5318.01to12175.24)	6.22 (3.97to8.75)	123.86 (79.33to178.63)

DALYs, disability-adjusted life-years; ASMR, age-standardized mortality rate; ASDR, age-standardized DALY rate; UI, uncertainty interval.
